# Pathogenesis and therapeutic implications of EBV-associated epithelial cancers

**DOI:** 10.3389/fonc.2023.1202117

**Published:** 2023-10-02

**Authors:** Yi Hua Low, Celestine Jia Ling Loh, Daniel Yang Yao Peh, Axel Jun Ming Chu, Shuting Han, Han Chong Toh

**Affiliations:** ^1^ Duke-NUS Medical School, Singapore, Singapore; ^2^ Lee Kong Chian School of Medicine, Nanyang Technological University, Singapore, Singapore; ^3^ Singapore Health Services Internal Medicine Residency Programme, Singapore, Singapore; ^4^ Division of Medical Oncology, National Cancer Centre Singapore, Singapore, Singapore

**Keywords:** Epstein-Barr virus, nasopharyngeal cancer, lymphoepithelioma-like carcinoma, primary pulmonary lymphoepithelioma-like carcinoma, pathogenesis

## Abstract

Epstein-Barr virus (EBV), one of the most common human viruses, has been associated with both lymphoid and epithelial cancers. Undifferentiated nasopharyngeal carcinoma (NPC), EBV associated gastric cancer (EBVaGC) and lymphoepithelioma-like carcinoma (LELC) are amongst the few common epithelial cancers that EBV has been associated with. The pathogenesis of EBV-associated NPC has been well described, however, the same cannot be said for primary pulmonary LELC (PPLELC) owing to the rarity of the cancer. In this review, we outline the pathogenesis of EBV-associated NPC and EBVaGCs and their recent advances. By drawing on similarities between NPC and PPLELC, we then also postulated the pathogenesis of PPLELC. A deeper understanding about the pathogenesis of EBV enables us to postulate the pathogenesis of other EBV associated cancers such as PPLELC.

## 1 Introduction

Epstein-Barr Virus (EBV) is a ubiquitous oncovirus that affects more than 90% of adults worldwide, but is especially endemic in East Asia, South East Asia, North Africa and Polynesia (1). The oncogenic potential of EBV is well established and yet evolving still, and its role has been demonstrated in the pathogenesis of epithelial malignancies such as Epstein-Barr virus- associated gastric cancers (EBVaGCs), nasopharyngeal carcinoma (NPC), lymphoepithelial like carcinoma (LELC), as well as in hematological malignancies such as Burkitt’s lymphoma, Diffuse Large B Cell Lymphoma (DLBCL), Hodgkin’s disease, and natural killer T-cell lymphomas (2).

While EBV-associated epithelial cancers vary in presentation and cell origin, these malignancies have in common a viral-mediated immune-suppressed tumor immune microenvironment, mutational signatures and epigenetic hallmarks (3). Among these epithelial cancers, the pathogenesis of NPC has been the most well described, in particular the role of EBV in transforming nasopharyngeal epithelium in a possible multi-step process from dysplasia to carcinoma (4). However, this process has not been described for other epithelial cancers, especially in lymphoepithelial like carcinoma (LELC).

We aim to discuss the pathogenesis of NPC and EBVaGC, and hypothesize the pathogenesis of LELC based on their common epigenetic, genomic and somatic mutational signatures. In particular, we examine primary pulmonary LELC (PPLELC) and consider the hypothesis of the EBV transformation of dysplastic epithelial cells in the lungs.

### 1.1 NPC

NPC is a head and neck cancer that is found commonly in Guangdong Province, Hong Kong, Southeast Asia, East Asia, and the Mediterranean area (5); however it is relatively uncommon in Western countries (6). A study in Singapore found that NPC has a higher incidence amongst Cantonese compared to the Teochew- or the Hokkien-dialect group (7). Similar findings were also observed in China, where the Cantonese ‘boat people’ in Southern China and provinces near Guangdong have a relatively higher incidence of NPC (8). This geographical clustering of NPC suggests that both genetic and environmental factors contribute to its development. Genetic factors, including HLA-A*0207 or B*4601 and disease-associated SNPs such as TLRs (which will be discussed in section 5) may increase an individual’s susceptibility to NPC. Furthermore, evidence of genetic susceptibility is also shown by the increased NPC risks in those with first-degree family members with NPC (9). Environmental factors, such as the consumption of volatile nitrosamine containing preserved foods and salted fish, are also strongly linked to NPC and are commonly found in the Cantonese diet (10).

According to epidemiological studies, NPC occurs two to three times more frequently in males than females (11). It has a bimodal distribution in low-risk populations, reaching a modest peak from ages 15 to 24 years old, and reaching a higher peak from ages 65 to 79 years old (11). In high-risk populations, NPC incidence peaks at 50 to 59 years old, and subsequently decreases (12).

The World Health Organisation (WHO) classifies NPC into three major groups, namely, the keratinizing squamous subtype (Type I), nonkeratinizing squamous subtypes (Type II), and undifferentiated or poorly differentiated (Type III). EBV and its association with the non-keratinising forms of NPC has been well-established (6). This is evidenced by the presence of EBV in dysplastic nasopharyngeal epithelium, as well as its clones in NPC biopsies in a few studies (13). Anti-EBV immunoglobulin A (IgA) has also shown utility as a screening marker for NPC, especially in patients with a strong family history (14). In addition, serum EBV capsid antigen (VCA) IgA or EBV DNA titers were also found to be associated with risk of NPC, with higher levels correlating with advanced disease (9, 15).

NPC is usually found in the lateral wall of the nasopharynx, in particular, the fossa of Rosenmuller. As the disease progresses, it can either remain confined within the nasopharynx, or extend into the contralateral lateral wall, skull base, palate, nasal cavity or oropharynx (16). While NPC most commonly presents with cervical lymphadenopathy (16), it can also present as cranial nerve palsies, blood in mucus or saliva, hearing loss, serous otitis media, tinnitus, diplopia, dysphagia and dysphonia, and occasionally pain (17). In the recently published 8th edition of the TNM staging for NPC by the American Joint Committee on Cancer (AJCC) (18), NPC is classified into 5 stages with stage 0 suggesting preinvasive lesion, I to II indicating early disease, and stages III and IV broadly indicating more extensive lymph node involvement, with IVB indicating metastatic disease.

### 1.2 Epstein-Barr virus-associated gastric cancer

EBVaGC constitutes 10% of gastric cancer (19) and is one of the most common EBV-associated malignancies by overall incidence. It is defined by the monoclonal proliferation of latent EBV-infected carcinogenic cells (20). Like NPC, EBVaGC is more commonly found in males, younger individuals (21). However, unlike NPC and PPLELC, the incidence of EBVaGC is higher in Western countries such as Germany and the United States as compared to Asian countries (22). EBVaGC is predominantly located in the proximal stomach and remnant stomach post partial gastrectomy for gastric ulcers or cancer (20). Clinically, EBVaGC presents with loss of weight, epigastric pain, early satiety, gastrointestinal bleed, iron deficiency anemia, and nausea (3).

Similar to NPC, consumption of salty food and exposure to wood dusts increase risk of EBVaGC (23–25). *H. pylori* infection, a significant risk factor for non-EBV gastric cancer, was not found to be associated with EBVaGC.

### 1.3 LELC and primary pulmonary LELC

LELC is a poorly differentiated carcinoma characterized by dense lymphocytic infiltration in the stroma, histologically similar to undifferentiated NPC. It is a rare but distinct cancer that may arise from multiple organs, including the lungs, stomach, parotids, salivary gland, thymus, biliary tract, breast, prostate, and metastatic NPC has to first be excluded (26, 27).

The first recognition of the possible association of LELC of the lungs with EBV was by Begin et al (28), and since then, the presence of EBV has also been detected in LELCs of the salivary gland (29), gastric (30, 31), thymus (32), colon (33), lung (34) and the intrahepatic biliary tract (35). The identification of EBV in LELC has led to an increasing amount of research exploring the role of the oncovirus in the pathogenesis of LELC (36).

PPLELC, or LELC of the lungs, is classified as a subtype of non-small cell lung cancer (NSCLC) and is the most common type of LELC. Similar to NPC, it is found more commonly in Asians, in particular the Southern Chinese (36–38), and when found in westerners, it is typically EBV negative (39–41). It presents at a lower mean age of 51-55 years old and has a better prognosis than non-LELC lung cancers (42), with a higher incidence in non-smokers and Asian females (43). Since its first documentation (28), current understanding of the histological resemblance, geographical clustering and molecular similarities (36) between NPC and PPLELC have prompted increasing interest in the role of EBV in PPLELC (34, 44, 45). Currently, PPLELC is the most well studied LELC subtype and will be the primary focus when discussing LELCs.

## 2 Pathogenesis of NPC

EBV is known to be one of the etiological agents of NPC pathogenesis (46). While lytic replication typically predominates during the acute phase of EBV infection and early dysplasia, persistent latent EBV infection, clonal expansion of infected epithelial and lymphoid cells are thought to be drivers of NPC (47), and the lytic-latent switch is an important step in malignant transformation.

EBV is potentially acquired in early childhood when the immune system is not fully developed and evolved (48). On the contrary, if acquired during teenage years, EBV potentially presents as infectious mononucleosis (49, 50). One hypothesis proposes that in early life, persistent EBV infection arises from inadequate immune clearance due to the developing immune system resulting in an infection that is likely to persist for the remainder of the carrier’s lifespan. Because of this, early infection is more likely to result in latency, which when exposed to genetic and environmental factors, is particularly susceptible to malignant transformation (51). In the case of NPC, EBV expresses a type II latency program expressing specific gene products that enhances its ability to survive and spread. This will be further elaborated in the later sections.

## 3 Pathogenesis of EBVaGC

One hypothesis of EBV pathogenesis in EBVaGC suggests that direct ingestion of the EBV virus could result in the infection of gastric epithelial cells (52, 53). It has also been postulated that during the reactivation of the lytic phase, resident B lymphocytes present in gastric mucosa release EBV, which subsequently infects more epithelial cells, a process thought to be aided by ephrin receptor A, integrins, and non-muscle myosin heavy chain IIA (NMHCIIA) (54). This process is further facilitated by upregulation of adhesion molecule-1 through integrin β1/β2 mediated contact between B lymphocytes and gastric epithelial cells and clathrin-mediated endocytosis (3). Congruent with this hypothesis, EBV infection is found in a small number of non-neoplastic gastric mucosa, suggesting that EBV infection precedes the monoclonal expansion of EBV-infected cells in tumorigenesis (55, 56). EBV anti-VCA and anti-EBNA antibody titres are also elevated in patients with gastric dysplasia on biopsy, indicating that EBV reactivation may be related to early tumorigenesis of EBVaGC.

After establishing infection in epithelial cells or B lymphocytes, EBV maintains a type I or II latency programme. This includes the expression of latent genes like EBERs, EBNA-1, Bam-HI A rightward transcripts (BARTs), miR-BARTs, and latent membrane protein 2A (LMP2A) (57, 58). EBER1 upregulates insulin growth factor 1, promoting the growth of EBVaGC (59). It also triggers chemoresistance and cell migration through the IL-6-STAT3 pathway (60), and facilitates tumorigenesis by blunting cellular responses to DNA damage through disruption of promyelocytic leukemia nuclear bodies (61). Furthermore, EBNA1 sequesters reactive oxygen species via miR-34a and NOX2, increasing cell viability (62). LMP2A was found to activate the nuclear factor kappa-light-chain-enhancer of B cells (NF-κb) pathway to upregulate miR-155-5p, resulting in the inhibition of p-Smad2 and Smad 2, inducing epigenetic alterations in the host genome (63).

## 4 Proposed pathogenesis of EBV in PPLELC

PPLELC is a poorly differentiated carcinoma with a rich lymphoid infiltration in its stroma, which is histologically indistinguishable from metastatic NPC. This raises the possibility of a “converging” role of EBV transformation in these epithelial cells, leading to a poorly differentiated subtype. Whilst the majority of LELC existing in case series are of PPLELC, gastric LELC (64), intrahepatic cholangiocarcinoma (ICC)-LELC (65) and parotid LELC (29), which suggest an aerodigestive epithelial nidus of pathogenesis, not all LELCs described originate from epithelial cells directly in contact with environmental carcinogens or EBV infection.

**Figure 1:**
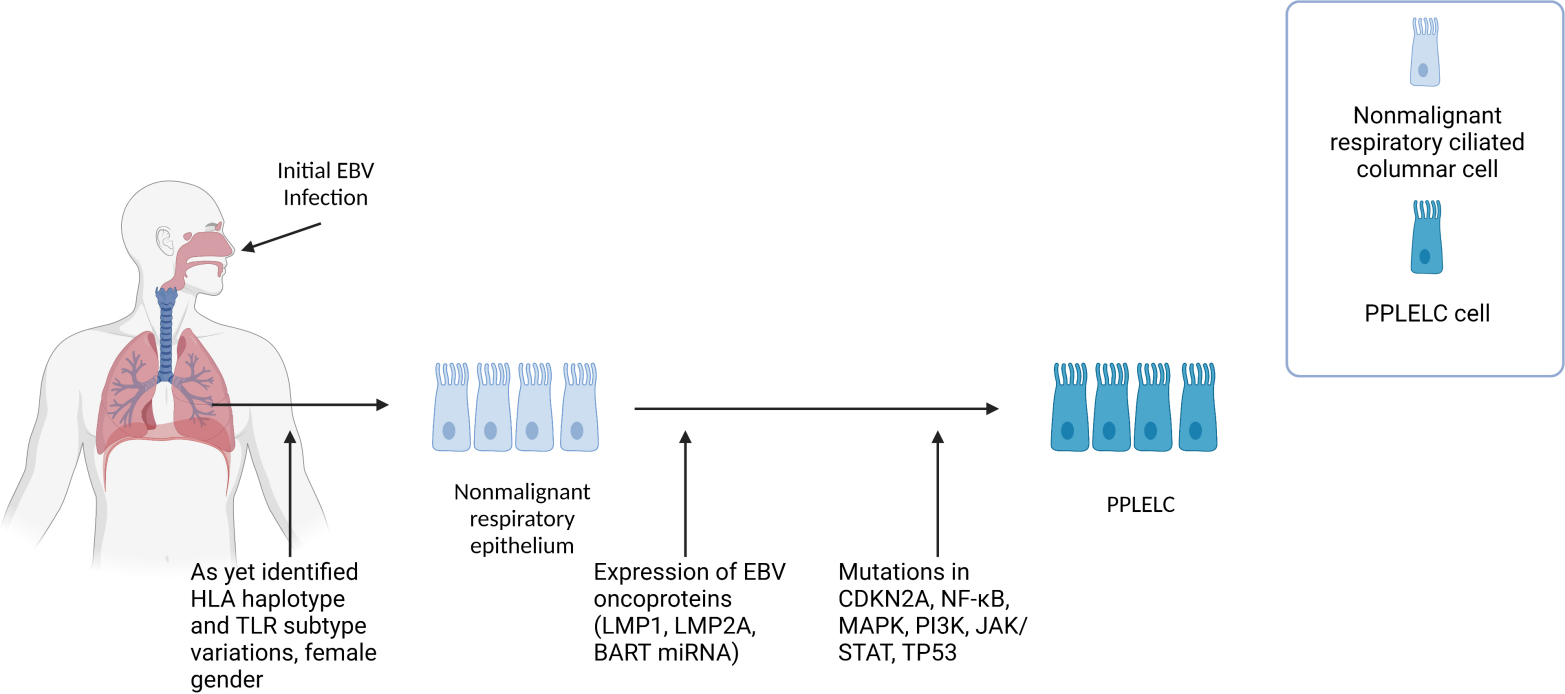
**Figure 1** describes the proposed multistep pathogenesis of PPLELC. We propose that PPLELC follows a multistep pathogenesis process similar to that in NPC. **Early events** Like NPC, distinct genetic profiles (unidentified HLA haplotype, and TLR subtypes variations) and early EBV infection may result in susceptibility to EBV infection. Early infection results in a type II latency program, suggested by a molecular profile that is highly resembling that of NPC. Additional risk factors such as being of a Chinese race, female, and nonsmokers are also associated with increased incidences of PPLELC. However, our knowledge of the role of epigenetic and environmental risk factors in PPLELC is still in its infancy. **Type II latency program** In these non-malignant respiratory ciliated columnar cells, EBV can establish latency and subsequently express latent EBV oncoproteins (LMP1, LMP2A, BART miRBA). Mutations in TP53, JAK/STAT, and cell cycle genes such as CDKN2A and CCND1 further drive non-malignant respiratory epithelium to PPLELC. The molecular and mutational landscape in PPLELC bears a striking resemblance to the molecular profile of NPC, suggesting that EBV enters a type II latency program in PPLELC. While it is unclear how these type II latency oncoproteins confer oncogenic traits in PPLELC, extrapolations can be drawn from our understanding of their established roles in NPC pathogenesis. It is likely that these oncoproteins and their downstream signaling pathways help enhance cell survival and facilitate tumorigenesis, resulting in the transformation from non-malignant respiratory epithelium to PPLELC.

At present, our understanding of how EBV contributes to PPLELC pathogenesis is limited, primarily relying on inferences drawn from our knowledge of EBV in the context of NPC, and similarities in the gene signatures and EBV latency in PPLELC. We propose that PPLELC adopts a similar model whereby a type II latency program in dysplastic tissue, accompanied by other predisposing factors, leads to the development of cancer (**Figure 1**).

Current literature has also established the presence of EBV viral load in PPLELC and its correlation to treatment response and prognostication (66–68). Here, we also posit the idea that early childhood latent infection of EBV in patients who develop PPLELC in later years possess underlying genetic predispositions such as variations in HLA-haplotypes and TLR subtypes, similar to the existing multistep model proposed in NPC (4). Subsequently, EBV oncoproteins and its downstream signaling pathways drive airway epithelium into malignant epithelial cancers (**Figure 1**). However, due to the rarity of this disease and its sparse literature, the genetic and environmental predispositions remain to be proven by genome-wide association studies and epidemiology studies.

## 5 Environmental carcinogens and genetic susceptibility of NPC, PPLELC, and EBVaGC

With the nasopharynx being located in the upper airway, and the lungs in the lower airway, both are exposed to similar viral infections and carcinogens; similarly, the nasopharynx and the upper digestive tract also share other carcinogens through inhalation and ingestion.

Several environmental risk factors have been found to increase NPC and EBVaGC risks. Dietary consumption of salt-preserved fish and preserved foods such as eggs, meat, and vegetables (12, 17, 69, 70) are highly associated with NPC. Exposure to wood dust (71–75), formaldehyde and solvents such as phenoxy acid and chlorophenol (76, 77) have also been linked to NPC and EBVaGC (23–25). Additionally, steam, flammable products, cotton dust, and smoking also increased NPC and EBVaGC risks (22, 78). A case-control study conducted among male patients in the Guangdong province demonstrated that a smoking pack-year of 40 or more predisposed an individual to a significant odds ratio of 1.76 to contract NPC. Similarly, 20-40 smoking pack-years was associated with a significantly increased risk of NPC, with an odds ratio of 1.52.

Histological and geographical similarities between NPC and PPLELC have prompted increasing comparisons between the two. Even though epidemiological studies on PPLELC are sparse, a recent study in Singapore revealed racial similarities, in which the Chinese race is more frequently affected (79). While still unexplored, a Chinese predominance may suggest that risk factors common in the traditional diet or environment of endemic regions could also be associated with PPLELC. While race is a similarity between PPLELC and NPC, there are some differences. For example, PPLELC tends to occur in non-smokers (43) and females (79). More studies are warranted to better describe the environmental risk factors associated with PPLELC.

Apart from environmental and epidemiological risk factors, certain genomic variants increase susceptibility to NPC. Several HLA alleles have been associated with increased risks of NPC, such as HLA-A1, HLA-A2, HLA-A*0207, HLA-A*33:03, HLA-B14, HLA-B*38:02, and HLA-B46 (80, 81). On the other hand, HLA-A11, HLA-A*11:01, HLA-A23, HLA-A*31:01, HLA-B*13:01, HLA-B14, HLA-B22, HLA-B27, HLA-B55, HLA-B*55:02, and HLA-DR4 were found to be protective factors against NPC carcinogenesis (80, 81). Variants in expressions and polymorphisms in toll-like receptors (TLR), a mediator of the immune system, were also found to increase the risk of NPC. Sequence variants in TLR10 and a functional variant in the 3’UTR of TLR4 were associated with increased NPC risk (82, 83). Genetic polymorphisms of TLR3 (84), TLR9 (85), and TLR4 (86, 87) were also linked to increased risks of NPC.

Compared to NPC, little is known about HLA and TLR variants in EBVaGC and PPLELC. While one study with 52 EBVaGC patients found no interactions between EBV and TLR polymorphisms (88), other recent studies have shown a reduced level of TLR9 in EBVaGC, suggesting its potential involvement in EBV reactivation (89). In PPLELC, TLR variants and HLA haplotypes conferring increased susceptibility have yet to be investigated, and is an avenue for future research.

## 6 Genetic and epigenetic characteristics in NPC, PPLELC, and EBVaGC

**Figure 2:**
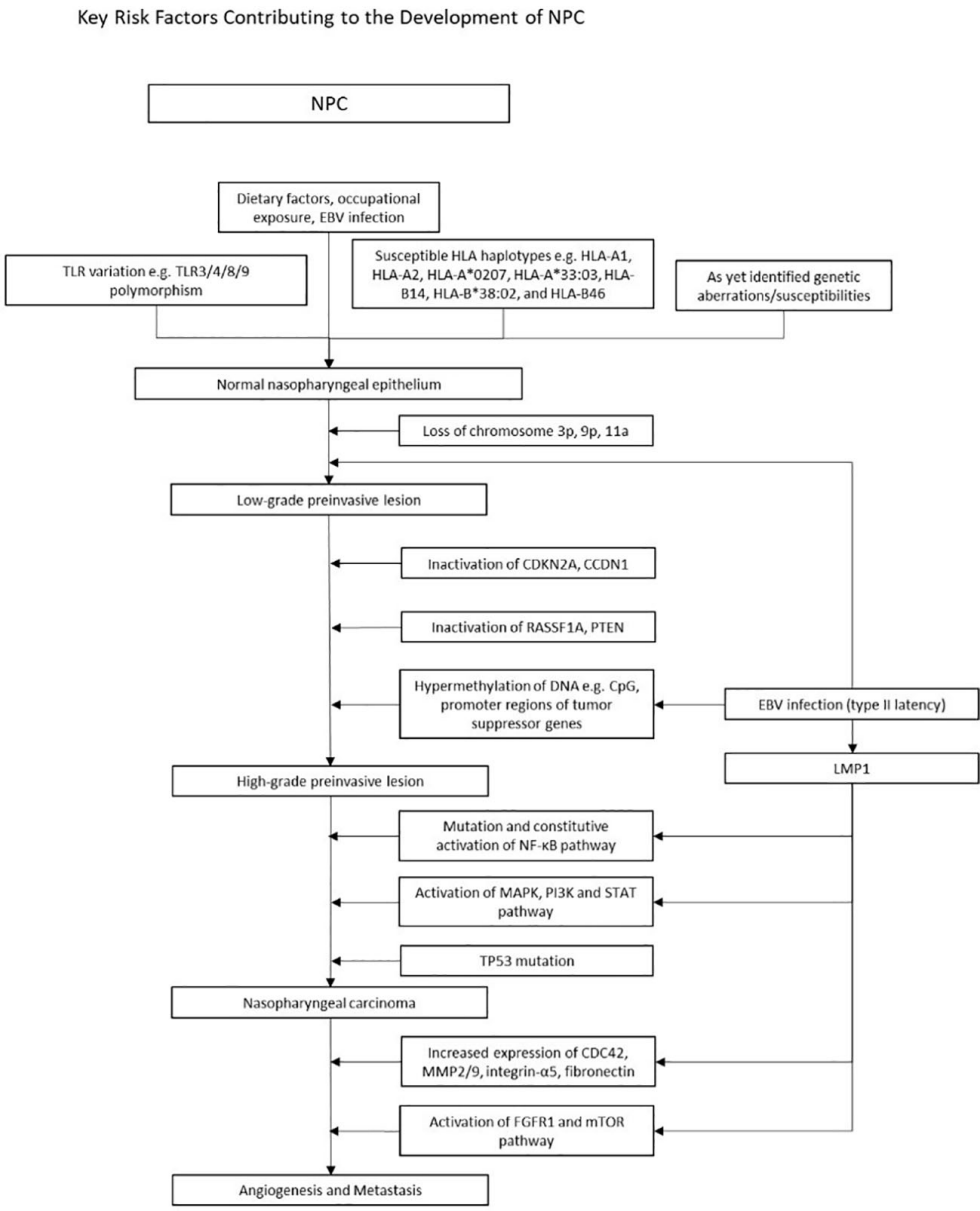
**Figure 2** illustrates the multistep model of the pathogenesis of EBV positive NPC. A complex interplay of known genetic variations with environmental risk factors increases susceptibility to NPC. Subsequent chromosomal loss (3p, 9p, 11a), loss of function mutations (CDKN2A, cCDN1, RASSF1A, PTEN), and widespread hypermethylation lead to high-grade preinvasive lesions. Mutations in the NF-kB, MAPK/PI3K/STAT and TP53 pathways further drive tumorigenesis. Concurrently, EBV oncogenes upregulate the expression of various genes, perpetuating tumorigenesis. **Early events in NPC pathogenesis and epigenetic changes** Numerous environmental factors have stood out in its relation to NPC. Dietary habits, such as preserved fish and foods, as well as occupational exposures to wood dust, formaldehyde and cigarette smoke emerged as significant contributors which heightened risk of NPC. In early stages of NPC, in addition to various TLR and HLA polymorphisms, epigenetic alterations such as CpG Island hypermethylation, and chromosomal loss (3p, 9p21) play a pivotal role in silencing tumor suppressor genes, contributing to disease initiation and progression. **Abnormalities in signaling pathways** Dysregulation of the NF-KB, MAPK PI3K and STAT pathways are well known in NPC. LMP1 activates NF-KB by engaging both the canonical and non-canonical pathway, facilitating apoptotic evasion and immune escape. The MAPK, PI3K and STAT pathways are also activated via the TNF receptor, upregulating anti-apoptotic genes and pro-survival signals. **LMP1 and tumorigenic properties** LMP1 is one of the key players of tumor progression, orchestrating various mechanisms that drive malignancy. Through activation of the FGFR1, mTOR and, the NF-KB pathway, upregulation of MMP, fibronectin and integrin-a5, and enhanced VEGF expression, LMP1 stimulates new vessel formation, a critical factor facilitating tumor proliferation and metastasis. Simultaneously, LMP1 disrupts immune surveillance by hampering antigen presentation and amplifying anti-apoptotic signals, allowing it to evade immune detection and circumvent cell cycle checkpoints. EBV fosters a tumor suppressive microenvironment and this is enabled by LMP1 upregulation of IL-10 and upregulation of T helper cells. Uncontrolled cell proliferation is attained through LMP1 hyperphosphorylation of DK2 and Rb thus promoting G1/S progression, and LMP1 regulation of telomerases resulting in cell immortality. Other prominent EBV products include LMP2 and EBNA1, which are further detailed under Figure 3. **An overview of the development of NPC** In summary, Figure 2 provides a brief overview of the multi step model of NPC pathogenesis. This model posits that the development of NPC is a highly complex, multistage process of cumulative environmental exposures and genetic changes, spanning from initial infection and environmental encounters leading to epithelial dysplasia, and subsequent genetic and epigenetic alterations over time activating oncogenesis. Products of type II latency program then confer tumorigenic properties to NPC cells, enabling growth and aggressive invasion.

In NPC, early events such as genetic susceptibility, environmental carcinogens and epigenetic alterations drive normal epithelium to dysplasia through chromosomal loss and EBV-related malignant transformation (4). Subsequently, further somatic mutations and/or epigenetic inactivation events (cell cycle checkpoint, TP53 and other TSG inactivation) lead to tumor progression (**Figure 2**). This process is accompanied by angiogenesis, metabolic dysregulation and increased invasiveness, resulting in metastasis and immune evasion.

Early studies suggest chromosomal aberrations that are detected in the dysplastic nasopharynx, including the loss of chromosomes 3p, 9p, 11q, and 14q (90–93), with the most widely known being 3p (95%) and 9p21 (85%) (4). Interestingly, these genetic deletions occurred even in the epithelium of people with no NPC in endemic regions, suggesting that these epigenetic changes occur early in disease, and may predispose individuals to EBV infection (91, 93, 94). Significant hypermethylation at chromosome 6p21.3 were found in both NPC and EBVaGC (95). This region contains numerous genes encoding for tumor suppressors and HLA genes that are known to be important risk factors in NPC (95, 96). Apart from the epigenetic alterations in tumor suppressor genes (TSGs), amplification of several oncogenes were also found in 8-20% of NPC. These include phosphatidylinositol-4,5-bisphosphonate 3-kinase catalytic subunit α (PIK3CA) and CCND1 on 3q26.3 and 11q13, respectively. The lymphotoxin-β receptor, a NF-κB signaller found on 12p13, was also amplified. Augmentations in PI3K-Akt and NF-κB signaling contribute to the tumorigenesis of NPC (97–99). Similar to NPC, mutations in PIK3CA are widely seen in EBVaGCs.

Genome sequencing and array-based methylomes have revealed a viral-mediated CpG Island hypermethylation Phenotype (CIMP) in NPC (95, 100, 101). EBV is known to induce a distinctive hypermethylated epigenotype in EBVaGC, NPC and LELC (63, 102–106), where important TSGs are inactivated by extensive methylation at the promoter region (107), in line with oncogenic behavior. Hypermethylation alters the balance between DNA methyltransferases and demethylases, maintaining type II latency. LMP1 and LMP2 are key players in promoting CIMP hypermethylation, which subsequently downregulates several TSGs such as Ras association domain-containing protein 2 (RASSF2A) (108), follistatin-like 1 (109), cyclin-dependent kinase inhibitor 2A (CDKN2A) (110), p16 (111), phosphatase and tensin homolog (PTEN) (112) and cadherin-1 (CDH1) (113), facilitating tumorigenesis and subsequent metastasis (94, 114). Although the pathogenesis of EBVaGC is not yet well defined, some have suggested that when EBV enters and establishes latency in the gastric epithelium, it undergoes genome wide methylation, a process similar to that in NPC (22, 115). The methylation of TSG (APC, PTEN and RASSF1A), cell adhesion molecules (THBS1 and E-cadherin), and CDKN2A are also widely found in EBVaGC (20, 116–119). Other methylated genes include MLH1, CXXC4, TIMP2 and PLXND1 (20).

Several molecular commonalities underlie PPLELC and NPC. Molecular profiling revealed similar LMP1 (120) and BART miRNA profiles between EBV positive PPLELC and NPC (121). Due to the expression of similar latent genes, it has been postulated that PPLELC and NPC share a similar pathogenesis involving a type II or III EBV latency programme, activating similar downstream pathways (122). Whole exome sequencing data from 30 patients with PPLELC showed that the mutational landscape of PPLELC closely resembled NPC, instead of other lung cancers or NKT cell lymphoma (123). NF-κB pathway genes, such as silencing mutations of negative regulators of NF-κB including TRAF3, were found in PPLELC, suggesting a similar oncogenesis pathway to NPC (122, 123). Likewise, mutations in TP53, JAK/STAT, and cell cycle genes such as CDKN2A and CCND1 were found in both NPC and PPLELC (116, 123, 124). High activation of AID/APOBEC genes (type 2 mutation signature), indicating a secondary immune response to EBV, is associated with the carcinogenesis of PPLELC (123), again pointing to the importance of EBV in shaping the somatic mutational landscape. While the tumor mutation load of PPLELC is low, large amounts of gene copy number changes, such as 11q13.3 amplification and 9p21.3 deletion were found, similar to the copy number variation in NPC (123).

EBV positive NPC has several unique traits which are distinct from other cancers, and these characteristics are reflected in PPLELC. Although a loss of function mutation of TSG p53 is present in many cancers, EBV associated NPC is known to lack this aberrance and usually have a normal profile of p53. Similarly, p53 levels were also found to be normal in EBV positive PPLELC tissues and with low levels of mutated p53 in PPLELC (122), suggesting a similar mechanism could be driving both NPC and PPLELC. This is similar to EBVaGC, where p53 mutations are rarely seen (125, 126).. EGFR activating mutations, ALK gene arrangement and ROS1 gene arrangement were also rarely found in PPLELC (127). Microsatellite instability is exceedingly rare in NPC (128), and this has been reflected in PPLELC (129). Also, the loss of heterozygosity at D5S346 (5q23) is common, suggesting that the pathogenesis of PPLELC is associated with a TSG in that region (129).

Existing models in NPC postulate a multistep pathogenesis process where early environmental exposures as well as distinct genetic profiles predispose an individual to EBV, and acquired epigenetic changes may result in susceptibility to EBV infection (**Figure 2**). Upon infection by EBV, latent genes expressed upregulate certain gene products which facilitate cell survival and malignant transformation, resulting in the further development of NPC (91). In view of its similarities to NPC as well as a similar model described for EBV related lung cancers (130), we suggest that PPLELC may adopt a similar multistep pathogenesis pathway (**Figure 1**). We hypothesize that EBV infection is an early transforming event, and that along with predisposing environmental risk factors, normal airway epithelial cells are transformed into poorly differentiated carcinoma.

## 7 EBV Latent Infection in NPC, PPLELC, and EBVaGC

EBV (human herpes virus type 4) is a double-stranded DNA (dsDNA) of approximately 170 KB. EBV is usually transmitted through the saliva (131) and infects both lymphoid and epithelial cells. Primary infection of EBV occurs as the virus enters the mucosal epithelium from the oropharynx and spreads to lymphoid tissues where it gains entry into the nasopharyngeal epithelial cells, facilitated by Ephrin A2 receptor and the vitronectin receptors αvβ5, αvβ6 and αvβ8 (132–134). Unlike in the latent phase where the EBV genome is only replicated once throughout the cycle, in the lytic phase, the EBV genome can undergo large numbers of amplification to generate numerous viral genomes which can infect other cells (81). While recent studies of the lytic phase and its involvement in tumorigenesis (135, 136) suggest that both phases might be involved in oncogenesis, the role of the latent phase in driving both lymphoid and epithelial tumors has been well established (91).

EBV enters a latent phase where the virus remains dormant in the nucleus and the carrier remains asymptomatic. EBV establishes latency, ranging from type I to III. In type I latency, latent genes such as EBNA1, EBER, and BART RNAs are expressed (137). In type III latency, nine proteins, namely (EBNA)-1, -2, -3a, -3b, -3c, and -LP, and (LMP)-1, -2a, and -2b are expressed. Of note, latency II gene expression is known to play a primary role in the pathogenesis of NPC (138), resulting in the expression of latent genes such as EBV nuclear antigen 1 (EBNA1), LMP1, LMP2, EBV-encoded small RNAs (EBER), Bam-HI A right frame (BARF) proteins, BARTs, and BART microRNAs (miR-BARTs). EBV effectively transforms and immortalizes B cells through various latent gene expressions such as LMP1, LMP2A (139) EBNA, BART and mir-BARTs (140), and a similar type II latency programme is suggested to exist in PPLELC (122). LMP1, LMP2a, and the NF-κB pathways were found to be involved in the carcinogenesis of PPLELC (122), suggesting that the pathway of tumorigenesis is likely to be highly similar (**Figure 1**).

### 7.1 Mechanisms of latent gene products in type II latency programme

**Figure 3:**
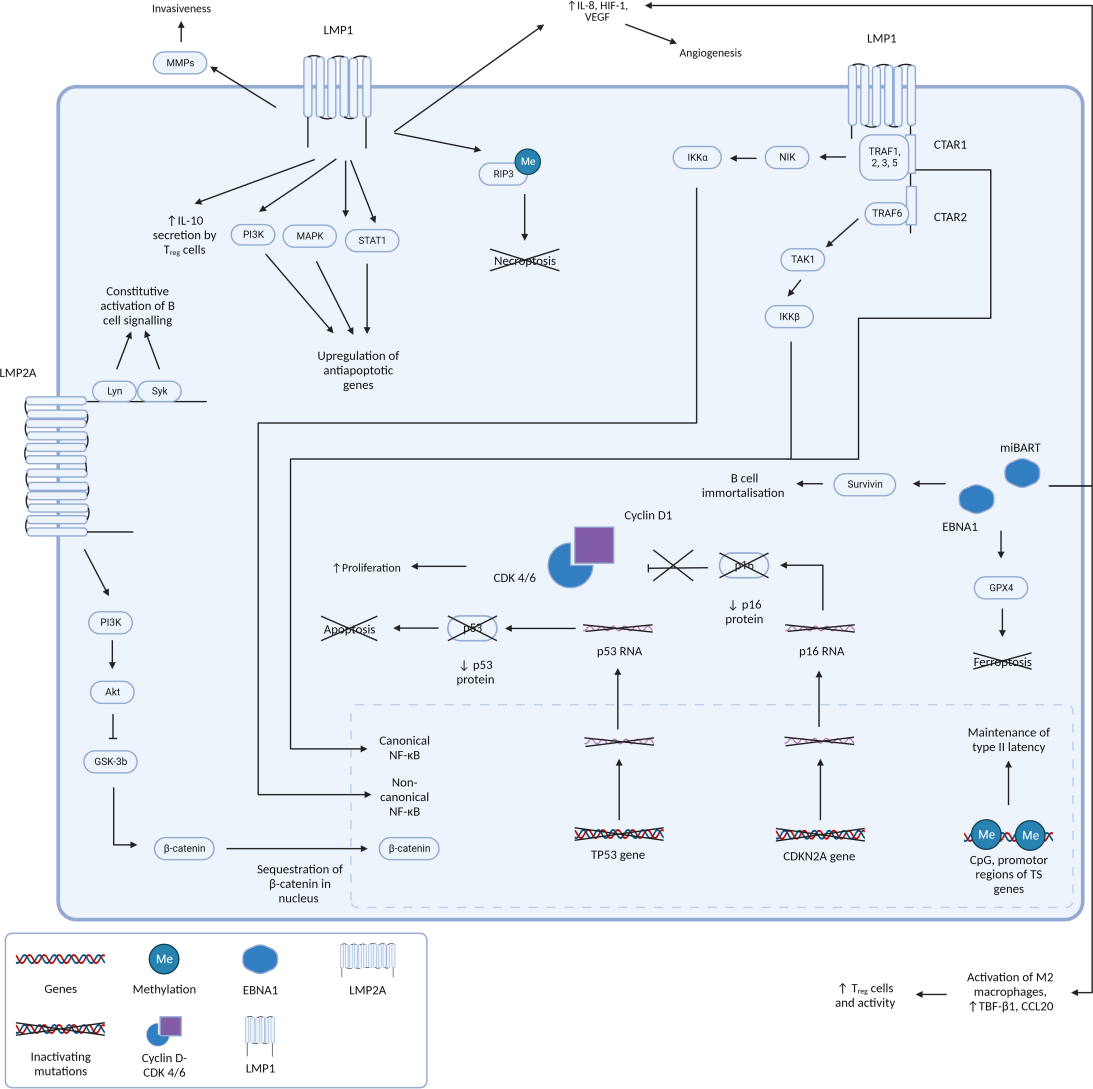
**Figure 3** presents a comprehensive and intricate depiction of the pivotal roles played by type II latency Epstein-Barr virus (EBV) oncoproteins, specifically Latent Membrane Proteins 1 and 2A (LMP1 and LMP2A), in conjunction with the crucial gene product EBNA1. It sheds light on how their orchestrated actions contribute to the initiation and progression of tumorigenesis by silencing of key tumor suppressor genes and driving cell proliferation and survival. **LMP1 and LMP2A: masters of oncogenic signaling** At the forefront of this dynamic interplay are the oncoproteins LMP1 and LMP2A. LMP1 is a multifunctional transmembrane protein. By emulating the constitutive activation of CD40, a pivotal B-cell receptor, LMP1 engages the TNF receptor-associated factors (TRAFs) and orchestrates the activation of the canonical NF-kB pathway. This activation culminates in the creation of a proinflammatory milieu that is conducive to cell survival and unbridled proliferation. Furthermore, LMP1’s capacity to activate the non-canonical NF-kB pathway further amplifies these signals, resulting in a sustained NF-kB activity that effectively shields cells from apoptotic cues. LMP1’s influence isn’t confined to NF-kB signaling alone. It interfaces with a multitude of molecules, including Janus kinases (JAKs), STATs, and PI3K, thereby activating pathways that foster cell growth and survival. Moreover, LMP1’s impact on various microRNAs and transcription factors significantly influences gene expression and cellular behavior. Beyond direct oncogenic signaling, LMP1 also modulates the tumor microenvironment, augment cellular invasiveness, and promotes angiogenesis. These help create a supportive niche for tumor progression and metastasis. Complementing LMP1, LMP2A emerges as a critical player in EBV-associated oncogenesis. This transmembrane protein mimics the B-cell receptor (BCR), a critical component in B-cell signaling. By recruiting protein tyrosine kinases, including Lyn and Syk, LMP2A effectively dampens BCR signaling. Activation of the PI3-kinase/Akt axis stimulates GSK3 which culminates in the accumulation of betacatenin, thereby promoting WNT signaling. This strategic inhibition aids in evading the regulatory mechanisms that would otherwise prompt apoptosis of autoreactive B cells. This evasion, in turn, promotes the survival of EBV-infected cells, and similar to LMP1, fosters a cellular milieu that supports enhanced proliferation and survival. **EBNA1: guardian of viral persistence and cellular transformation** Central to this network of interactions is the gene product EBNA1. This multifaceted protein stands as a linchpin in ensuring the persistence of EBV within the host cell. In addition to its role in maintaining viral genome replication, EBNA1 actively engages cellular machinery to promote cell proliferation and survival. Importantly, EBNA1 orchestrates the silencing of key tumor suppressor genes, notably CDKN2A and TP53. This results in an imbalance that shifts the cellular equilibrium toward unchecked cell growth and survival, fueling the malignant transformation of host cells. **A holistic view of tumorigenesis** Collectively, Figure 3 encapsulates the multifaceted orchestration of type II latency EBV oncoproteins, LMP1, LMP2A, and EBNA1, in driving the molecular intricacies of tumorigenesis. Their intricate molecular interplay, ranging from direct oncogenic signaling to the modulation of the tumor microenvironment, lays the foundation for cell transformation and the subsequent development of EBV-associated malignancies.

The involvement of latent gene products in NPC pathogenesis has been extensively documented and understanding its oncogenic properties and pathways in NPC allow us to hypothesize the pathogenesis and characteristics of other EBV positive tumors with a similar molecular profile, such as LELC. **Figure 3** summarizes the EBV gene products and molecular pathways described below which are known to be involved in NPC.

Unlike EBVaGC where mutations in the NF-κB pathway are uncommon (3), NF-κB pathway dysregulation is known to occur in EBV positive NPC (141). LMP1 is a key oncoprotein in NPC tumorigenesis and is known to establish multiple oncogenic hallmarks in the dysplasia-carcinoma model. It is a transmembrane protein that activates NF-κB through its C-terminal activation regions (CTAR1 and CTAR2) via the canonical and non-canonical pathways. These signaling domains mimic CD40, constitutively activating NF-κB. NF-κB transcription is upregulated through the canonical and non-canonical pathway. In the canonical pathway, CTAR2 recruits TRAF6/Transforming growth factor-β-activated kinase 1 (TAK1)/inhibitor of nuclear factor kappa-B kinase subunit β (IKK-β), and CTAR1 activates NF-κB through the non-canonical pathway, by recruiting TRAF1, 2, 3, 5, NF-κB-inducing kinase (NIK), and IKK-α (3, 142–146). CTAR1 also independently activates the canonical pathway of NF-κB (147). The LMP1 mediated canonical and non-canonical mediation of NF-κB pathways confer a survival advantage to tumor cells by enabling apoptosis evasion, B-cell activation, and survival (142), and is important for tumorigenesis via many pathways. Recent studies have also elucidated a role of NF-κB in regulating viral gene transcription of BART miRNAs and long noncoding RNAs, modulating the shift between latent and lytic states (124, 148), as well as promoting somatostatin receptor 2 (SSTR2) induction (149).

Apart from modulating the NF-κB pathway, LMP1 also functions as a constitutively active tumor necrosis factor (TNF) receptor, activating several other signaling pathways, including mitogen-activated protein kinase (MAPK), PI3K, and signal transducer and activator of transcription (STAT), resulting in the upregulation of anti-apoptotic genes (150). LMP1 also promotes stem-cell like properties, which are further maintained by the upregulation of oncogenes and anti-apoptotic genes like BCL2 and LEF1 (141).

LMP2A is a viral-encoded mimic of the B-cell receptor (BCR), and constitutively activates BCR-like signaling through recruitment of the Lyn and Syk kinases (151). It also provides a counteractive effect by depriving the BCR-signaling complex of its components. These two counterbalancing activities help EBV stay latent in B cells, evading detection by the immune system (152).

EBNA1 helps maintain the presence of the EBV genome in NPC as circular DNA episomes by binding to viral DNA. EBNA1 was found to be critical for the immortalization of B cells (153, 154). Survivin, an inhibitor of apoptosis, was also found to be activated in B lymphocytes by EBNA1 (155). In a recent study, it was demonstrated that EBNA1 results in a dose-dependent breakage at chromosome 11q23 (156). This is consistent with the fact that latent EBV reactivation has been associated with tumorigenesis, especially NPC (157). Similar to NPC, EBNA1 is also consistently expressed in EBVaGC (158) where it induces the loss of promyelocytic leukemia (PML) nuclear bodies (NBs) (61). This decreases p53 activation and apoptosis in response to DNA damage, allowing for EBVaGC carcinogenesis.

BART transcripts are abundant in NPC cells (121, 159), and are subsequently processed to miR-BARTs. miR-BARTS stabilize latent infection, suppress lytic replication by targeting BZLF1 and BRLF1 (159), downregulate apoptosis (160), and activate pathways promoting tumorigenesis (161).

While non-exhaustive, EBV latent gene products also trigger downstream pathways known to be involved in malignant transformation. For instance, aberrance in Wnt signaling regulation in NPC has been demonstrated by gene expression profiling studies (162). While studies have found that LMP1 does not play a critical role in direct activation of the WNT pathway (163), other latent gene products such as LMP2A activates PI3K/AKt to inhibit the downstream GSK-3b of Wnt, sequestering b-catenin in the nucleus and inducing EMT (164). Both LMP1 and LMP2A were also found to induce stem cell-like properties in NPC cells through Hedgehog signaling (165, 166). TGF-B1, a growth suppressor, is also inhibited by LMP1 (167). LMP1 also induces the expression of IL-10 and vIL-10, an immunosuppressive cytokine whose levels are raised in many cancers (168). Not only do the EBV latent gene products independently activate several downstream pathways beneficial for tumorigenesis, they also positively regulate one another leading to further upregulation (169, 170).

Interestingly, NPC and PPLELC have highly similar molecular landscapes. Like NPC, PPLELC also expresses high levels of LMP1, LMP2a, and NF-κB (122), with LMP1 detected in 42% of PPLELC (122). While little is currently known about how these gene products promote oncogenesis in PPLELC, these pathways have been well documented in NPC. Understanding how these common pathways foster angiogenesis and metastasis, cell cycle survival, and immune evasion in NPC, and also in other epithelial cancers such as EBVaGC, may guide our understanding in how these gene products establish tumor hallmarks in PPLELC.

### 7.2 Angiogenesis and Metastasis in NPC and EBVaGC

Angiogenesis is a major contributor to oncogenesis and metastasis as new blood vessel formation enhances nutrient and oxygen delivery to rapidly proliferating cells. In NPC, EBV latent proteins (EBNA1 and LMP1) promote angiogenesis through upregulation of angiogenic cytokines IL-8, hypoxia inducible factor-1 (HIF-1), and VEGF (171–173). LMP1 activation of NF-κB leads to the activation of the STAT pathway via increased levels of IL-6, and IL-8, promoting cell proliferation (174, 175), and angiogenesis (176) respectively. The role of EBNA1 in B cell transformation was also demonstrated in mice models where EBNA1 expression was associated with increased primary tumor formation and metastasis (177). The enhanced angiogenic ability can also be induced in neighboring endothelial cells promoting angiogenesis by the transferring of EBV encoded small RNAs and subsequent induction of vascular cell adhesion molecule 1 (VCAM-1) expression in endothelial cells (178). In EBVaGC, hypoxia-induced ebv-cir LMP2A promotes angiogenesis through the KHSRP/VHL/HIFa/VEGFA pathway (179). EBVaGC also upregulates IHH, a gene which increases metastatic potential through angiogenesis, Snail protein expression, and decreases e-cadherin and tight junctions (115). A loss of PTEN in EBVaGC activates PI3K/Akt pathway, resulting in increased angiogenesis, migration and loss of cell cycle adhesion (180). Downregulation of e-cadherin through ARID1A loss also leads to enhanced tumor migration and lymphovascular invasion (180). The WnT pathway is also downregulated by LMP2A in EBVaGC, enhancing cell migration and invasion (181). In both EBVaGC and NPC, EBV was found to promote vasculogenic mimicry formation through the PI3K/AKT/mTOR/HIF-1α pathway (182).

Matrix metalloproteinases (MMPs), key proteins responsible for the degradation of collagen IV, enabling basement membrane invasion, are also increased in EBV positive NPC and EBVaGC (183–186). LMP1 modifies its extracellular vesicles by increasing gene expression of MMP9 and MMP2 (187), and enhances cell attachment through the upregulation of integrin-α5 and fibronectin (187). Apart from directly modulating MMP expression, LMP1 also mediates other pathways such as cell division cycle 42 (CDC42), a protein known to regulate invadopodia formation (188, 189), actin cytoskeleton reorganization, and increase motility (190). Crucial cell adhesion molecules such as E-cadherin were also found to be downregulated by miR-200, miR-BART9 and LMP2A, BARF0, EBER and EBNA1 (191–193). The converse was found to be true, whereby a deletion in miR-BART9 results in elevated levels of E-cadherin expression, and a reduction in the proliferative and invasive potential of EBVaGCs (194).

A switch from oxidative phosphorylation to aerobic glycolysis is also beneficial for cell proliferation and invasion by providing intermediary metabolites (195). LMP1 activates glycolysis through the fibroblast growth factor receptor 1 (FGFR1) signaling pathway (196) and the mTOR pathway through auto-secretion of insulin-like growth factor 1 (IGF-1) (197). The predilection for aerobic glycolysis was evidenced by increased lactate levels, extracellular acidification ratio and oxygen consumption ratio suggestive of enhanced glycolysis, as well as reduced oxidative phosphorylation enzymes (197, 198). Glycolysis has also been linked with higher invasiveness as glycolytic enzyme expression was increased in cells with higher invasive properties than those without (197). Furthermore, the acidic tumor microenvironment due to lactate production also favors an environment for tumor cell migration (196, 199).

### 7.3 Cell cycle survival in NPC and EBVaGC

Tumour cells gain proliferative advantage by expressing aberrant genes which hijack regulation checkpoints, enabling hyperproliferation and apoptotic escape. In EBV-associated NPC, the CTAR1 domain of LMP1 hyperphosphorylates CDK2 and Rb, promoting G1/S cell cycle progression and protecting the cell from apoptosis (200, 201). Furthermore, LMP1 also regulates telomerase activity through the p16^INK4a^/Rb/E2F1, PI3K-AKT and c-Jun N-terminal kinase (JNK), promoting immortalisation (202). In fibroblasts, LMP1 suppresses p16^INK4a^ and p21WAF1 by H-ras61L, suppressing cell senescence (203). In EBVaGC, there is an upregulation of Bcl-2 and CyclinD1, allowing apoptotic evasion and progression of cell cycle through the G1 phase respectively (204). LMP2A plays a regulatory role in modulating the expression of cyclin E and the ratio of cells in the S phase (205) and inhibits TGF-β1 induced apoptosis (206, 207) miR-BART9, 11, 12 also reduced Bim expression, a pro-apoptotic protein (208).

Similar to other cancers, multiple cell cycle checkpoints (e.g. p16 and p21) are compromised by EBV. p16 is a TSG (CDKN2A) which inhibits cyclin D1/CDK4, and is commonly hypermethylated and silenced in NPC and EBVaGC (111, 114). Knocking out p16 expression, or over expression of its negatively regulated target cyclin D1/CDK4, promoted expansion of EBV infected cells (209). LMP1 also inactivates p16 by inducing sequestration of E2F4/5 and ETS-2 in the cytoplasm, rendering p16 dysfunctional in these cells (210). p21, another cell cycle inhibitor, was also downregulated in NPC cell lines by the binding of miR-17-5p to its 3’ untranslated (3’UTR) region (211). EBV positive NPC tumors are also more resistant to ferroptosis through EBNA1 upregulation of glutathione peroxidase 4 (GPX4) (212), and LMP1 inhibited necroptosis through RIP3 hypermethylation (213).

Interestingly, while p53 mutations, like other TSGs, are classically present in most other cancers, NPC has a unique profile of p53. Unlike other cancers which usually have mutations or loss of p53, there is little association between p53 mutations with NPC, and normal or elevated levels of p53 are often present (214–217). LMP1 levels positively correlate with p53 levels, and can induce p53 expression via the H19/miR-675-5p axis (218). Despite normal or elevated levels of p53, rapid proliferation suggests that EBV encoded products suppress the function of p53 instead (219). For instance, p53 expression can be inhibited by EBV encoded miR-BART5-3p, a loss of p14, as well as excess p63 (219–221). A similar pattern is also seen in EBVaGC where p53 mutations are rare (125, 126, 222), again suggesting that EBV encoded products suppress p53 function instead. For instance, BART3-3p and BART 5-3p bind TP53 to reduce senescence of EBVaGC, accelerate cell cycle progress, and prevent apoptosis (219, 223). It has also been suggested that normal or high p53 levels may confer a survival benefit as these cells will not be susceptible to JNK and Rapamycin-induced apoptosis (224, 225). Taken together, these suggest that p53 mutation does not have a major role in early pathogenesis (226), and subsequent p53 loss may occur as an independent event.

### 7.4 Immune modulation and evasion in NPC and EBVaGC

EBV is known to persist and escape immune clearance, and its presence induces an immunosuppressed tumor microenvironment (TME). Even though inflammation occurs in NPC (227), much of the TME remains immunosuppressed. While the latent phase was thought to be responsible for NPC pathogenesis, recent studies have also elucidated a role of the lytic phase in pathogenesis (135, 136).

In LMP1 positive NPC cells, IL-10, an immunosuppressive cytokine which reduces antigen presenting cells and T helper cell activity, was also found to be fourfold higher (228). LMP1 also plays an important role in creating an immune-suppressed TME by inducing interleukin-10 (IL-10) secretion in regulatory T cells. EBNA1 stimulated regulatory T cell (Treg) chemotaxis towards the NPC TME, through the upregulation of the transforming growth factor-β1 (TGF-β1)-SMAD3-PI3K-AKT-c-JUN-CXCL12-CXCR4 axis and downregulation of miR-200a (229). EBNA1 also upregulated TGF-B1 and CCL20 which converted naive T cells into Treg cells and increased Treg cells migration, respectively (230). Additionally, EBNA1 also activates M2 macrophages which encourages conversion of naive T cells into Treg cells (230). These are congruent with the presence of elevated levels of Treg cells found in the TME of NPCs (231) as well as its positive correlation to plasma EBV levels of NPC patients (230).

In EBVaGC, increased levels of Tregs, IL-1B, and IL-10 in EBVaGCs suggest an immunosuppressive environment (232, 233). This is evidenced by the impaired function of cytotoxic T lymphocytes (CTLs) and natural killer cells in EBVaGC. Elevated levels of IFN-γ depletes tryptophan and suppresses tryptophan sensitive cytotoxic T lymphocytes (CTLs) and natural killer cells (234). Additionally, LMP2A mutations on exons 1-8 (30) and PD-L1 upregulation in both EBVaGC and NPC (235–237) limit CTL detection and promote differentiation of CD4+ cells into Treg cells (238). Other molecules contributing to immunosuppression include CCL22 which increases Treg recruitment, and indoleamine 2,3-dioxygenase (IDO1) (234, 239, 240). Antigen processing in EBVaGC is also inhibited by EBNA1 repeats and early lytic gene BNLF1a (234, 241), perpetuating immune evasion.

## 8 Treatment options for NPC, PPLELC, and EBVaGC

Stage 0 and I NPC are usually treated with radiotherapy to the site of disease, with prophylactic radiation to cervical lymph nodes (242). Patients with stage II, III, and IV NPC are treated with concurrent chemoradiation (chemoRT), with stage III and IV NPC requiring either induction or adjuvant chemotherapy on top of chemoRT, usually cisplatin or cisplatin with fluorouracil (5-FU) or adjuvant oral capecitabine (243). Recurrent or metastatic NPC can be treated with palliative radiotherapy, chemotherapy, targeted therapy and/or immunotherapy. On the other hand, no clear treatment guidelines for PPLELC have been outlined at present (34). In general, surgical treatment is the mainstay of treatment for early PPLELC, and treatment options like chemotherapy, radiotherapy are usually reserved for more advanced stages of PPLELC (45). Because PPLELC is uncommon, the adoption of standardized treatments has been challenging. Existing treatment regimes are adapted from those used for stage IV NPC, which involves gemcitabine-based chemotherapy (45), taxanes (244), anti-angiogenic therapy (245), and anti-PD1/PD-L1 therapy (246). The favorable response of PPLELC to NPC treatments is evident of the therapeutic implications of molecular similarities between the two cancer types, and the substantial overlap in their molecular characteristics provides an explanation for the effective response of PPLELC to chemotherapies typically used in NPC. For EBVaGCs, early stage cancers are treated surgically via endoscopic mucosal resection or gastrectomy. The treatment options for locally advanced EBVaGC is similar to that of other gastric cancers. This includes perioperative chemotherapy with primary resection. Advanced EBVaGCs are treated with palliative chemotherapy (taxanes, irinotecan, platinums, and fluoropyrimidines) and VEGF-R antagonist ramucirumab (247).

The role of immunotherapy in the treatment of NPC has been increasingly explored and this is especially so because the tumor microenvironment of EBV-driven NPC creates conditions favorable for immunotherapy. Much attention has been placed on immune-checkpoint inhibitors (248). In 44 patients with recurrent or pretreated metastatic NPC, patients receiving nivolumab achieved a 1-year overall survival rate of 95% and 1-year-progression free survival of 19.3% (249). In the KEYNOTE-028 study (250), 27 patients with PD-L1 positive unresectable or metastatic NPC achieved a 25.9% objective response rate of partial response and stable disease. The more recent JUPITER-02 study demonstrated that the addition of toripalimib to the standard first line chemotherapy treatment of gemcitabine and cisplatin yielded a significant increase in progression-free survival (HR = 0.52, 11.7 v.s 8.0 months) and overall response rate (77.4 v.s 66.4%) (251). The addition of camrelizumab to the standard gemcitabine and cisplatin chemotherapeutic agents in the CAPTAIN-1st trial also demonstrated a significantly increased progression-free survival (HR = 0.54, 9.7 v.s 6.9 months) (252). In RATIONALE-309, the addition of tislelizumab to standard chemotherapy resulted in a significantly improved progression-free survival (HR = 0.50, 9.6 v.s 7.4 months) (253). However, the efficacy of immunotherapy does not extend when it is used alone as KEYNOTE-122 showed no improvement in overall survival when pembrolizumab was used as a monotherapy as compared to the standard chemotherapy agents used for recurrent or metastatic NPC (254).

Similar to NPC, PD-1/PD-L1 inhibitors are potential therapeutic options in PPLELC (255).. A recent study demonstrated that the use of sintilimab, pembrolizumab, and nivolumab in patients with advanced PPLELC helped achieve stable disease in 60% of the patients (255). The use of nivolumab resulted in partial remission in 36.4% of the patients, and stable disease in 54.5% of the patients. While the predictive value of PD-L1 as a biomarker of immune checkpoint inhibitor remains debatable (256, 257), a recent study by Zhong et al. demonstrated that higher PD-1/PD-L1 and LAG3 levels were indicative of a significantly better progression-free survival in a retrospective study (19 months v.s 3.9 months) (257).

Immunotherapy such as pembrolizumab have also demonstrated efficacy in EBVaGC as one study demonstrated a 100% (6/6) response to pembrolizumab in patients with EBVaGC (258). However, given the small sample size of that study, it is necessary to validate this finding with larger studies. The positive response of immunotherapy could suggest a potentially synergistic effect when administered alongside chemotherapy in EBVaGC. Though not shown yet, this is currently seen in advanced gastric cancer patients where the combination of chemotherapy and immunotherapy such as pembrolizumab and nivolumab was superior to using chemotherapy as a single agent (259), as well as in NPC as seen in the JUPITER-02 study.

Apart from immunotherapy, another treatment option is to employ cytotoxic T lymphocytes (CTLs). EBV-CTL is an autologous T-cell therapy generated through irradiated EBV-transformed lymphoblastoid cell lines (LCL). It was found that infusion of autologous EBV-CTL post lymphodepletion had efficacy as an anti-tumor agent for patients with locoregional NPC. In a recent phase III trial (VANCE trial, ESMO 2022 Oral presentation) in patients with recurrent or metastatic NPC, EBV-CTL administration following gemcitabine and carboplatin (GC) treatment also did not reveal superior efficacy over first line standard of care GC. Still, in the VANCE trial, improved survival trends were seen in a subset country specific analysis of overall survival and progression free survival for Taiwan, United States, and Singapore (unpublished). EBV-CTL may still be a viable treatment option as CTLs specific for LMP2 were shown to be associated with a significantly higher overall survival in a phase II study (260). EBV-CTL also demonstrated a very favorable safety profile (261). Like NPC, EBV-CTL also displayed efficacy in the treatment of PPLELC. In an unpublished study, our group was able to demonstrate complete response to EBV-CTL in a patient with heavily pre-treated advanced PPLELC in combination with an immune checkpoint inhibitor after previous progression on immune checkpoint inhibitor after just a few months.

A phase II trial in patients with locally recurrent NPC found that patients with Endostatin, an inhibitor of angiogenesis, administered in addition to cisplatin and paclitaxel had longer progression free survival with increased disease control rates (262). It was also found that anti-angiogenic therapy on top of radiotherapy improved clinical outcomes (263). Similarly, angiogenesis inhibition was also efficacious in PPLELC. In one study, small molecule multi-targeted TKI, inhibiting VEGFR, platelet derived growth factor (PDGFR), FGFR, and c-KIT was used to treat a patient with FGFR3-TACC3 fusion, and response was favorable. While studies are limited, this suggests that anti-angiogenic therapy can potentially be employed in the treatment of PPLELC (245).

Targeting EBV latent proteins has also shown potential as a viable therapeutic option. Not only is EBNA1 the only viral protein expressed in all EBV infected cells regardless of latency type, its vital role in maintaining EBV episomal genome and its consistent expression of EBNA1 in all tumor cells makes it a promising therapeutic target (47). Administration of conditionally replicating adenovirus expressed in an EBNA-1-dependent manner (adv.oriP.E1A) in EBV-positive NPC cells demonstrated cytotoxic effects and tumor regression in xenografts models, albeit limited clinical potential due to liver sequestration (264, 265). Preventing DNA binding of EBNA1 has also been an area of study. In a high-throughput virtual screen, several small molecule inhibitors, such as LB2, LB3, LB7 and LC7, SC7, SC11, SC19, SC27) (266) have been identified to selectively inhibit EBNA1 DNA binding (267). Others have also developed molecules that could inhibit EBNA1 DNA binding in xenografts, such as 2,3-disubstituted benzoic acid (268), of which one of its inhibitors is in a Phase/I/IIa clinical trial (269) (NCT03682055) in EBV positive NPC (47). Apart from targeting EBNA1, inhibition of LMP1 targeting DNAzyme found lower short-term tumor regression rate in a clinical trial of 40 patients with NPC (270).

Lytic inducers have the benefit of sensitizing tumor cells to anti-virals via the expression of viral kinases (271) during EBV reactivation. During reactivation, highly immunogenic viral replication proteins and virions are expressed which results in a strong immune response against EBV reactivated tumor cells (272). Because of this, oncogenesis is largely dependent on latency, and therapeutic strategies have been devised to inhibit latent genes or activate lytic genes with the aim of inducing oncolysis. For instance, it was found that transfection of EBNA-driven CMV BZLF1 plasmid induced BZLF1 reactivating EBV, resulting in cell death (273). Other compounds, such as histone deacetylase inhibitors (HDACis) or protein kinase C (PKC) activators have also been used to activate EBV lytic genes, albeit with varying effectiveness (274–277). This variability is likely attributed to the highly cell-dependent sensitivity towards lytic inducers, which also explains the diverse responses observed in oncolysis following the administration of gemcitabine and valproic acid in Phase I/II trials (278). Somatic mutations in TP53 and TGFBR2 were also found to maintain EBV latency in NPC (122, 279, 280), and may affect a patient’s response to lytic-inducer treatment.

The goal of understanding the intricacies of cancer pathogenesis is to intervene as upstream as possible, thereby thwarting the progression into dysplastic changes. Mitigating dysplasia and minimizing exposure to risk factors play pivotal roles in substantially lowering cancer risk. Consequently, there has been increasing focus on advancing vaccine development, implementing nuanced screening methodologies, and cancer biomarker development as part of a comprehensive preventative approach. Patients with NPC were found to have persistently elevated plasma EBV DNA (281) and plasma EBV is increasingly used as a screening tool for NPC (9) and a surveillance biomarker for patients in remission (282). Posttreatment plasma EBV DNA is the strongest independent predictor for cancer recurrence and long term survival, with high EBV DNA levels strongly correlating with disease progression, and low plasma EBV DNA posttreatment correlating with high progression-free survival rate at 2 years (283). Because EBV insidiously results in disease, therapeutic EBV vaccines can be highly efficacious and cost effective in lowering disease recurrence (284). The majority of current vaccines currently target EBV latent proteins such as EBNA1, LMP1, LMP2 (284). Several trials have also explored the potential of chimeric antigen receptor T cell (CAR-T) and T cell receptor-engineered T cell therapy (TCR-T) immunotherapy, with the latter more effective for solid tumors (284).

With EBV being a commonality behind the pathogenesis of EBV related epithelial cancers, we speculate the possibility of a universal off-the-shelf cell therapies targeting EBV-driven cancers and diseases, and this is an avenue for future research. This is especially relevant since recent studies noted clinical improvement in EBV-CTL treated EBV-driven MS, in which B-cells are proposed to be involved (285). With PPLELC and NPC sharing similar biological and molecular characteristics, it is unsurprising that treatments involving the same therapeutic targets have shown efficacy.

## 9 Discussion

Of the epithelial cancers associated with EBV, NPC and PPLELC have a predominant distribution in South China and Southeast Asia compared to the western population. On the other hand, EBVaGC is more prevalent in the western countries such as Germany and the United States (15-18%) (22). The oncogenic properties of EBV in NPC are well documented, and its latent genes LMP1, LMP2A, EBNA1, and BART transcripts, as well as its lytic genes, such as BZLF1, are known to participate in multiple pathways promoting tumor growth and metastasis, as well as imparting immuno-evasive characteristics. An overview of the characteristics of NPC, EBVaGC and PPLELC is shown in **Table 1**. While the connection between EBV and NPC has been well documented, its precise mechanisms remain elusive.

**Table 1:** Overview of the key similarities and differences between NPC, PPLELC, and EBVaGC.

	NPC	PPLELC	EBVaGC
**Etiology**	EBV infection	Postulated to be associated with EBV infection	EBV infection
**Epidemiology**	Rare in Western Countries, and more commonly found in Guangdong province of China, Hongkong, Southeast Asia, East Asia, and the Mediterranean area Males > Females	Rare in Western countries, commonly found in Asian countries like South China Females > Males	More common in Western countries like Germany and the United States Males > Females
**Risk Factors**	Smoking and preserved food containing volatile nitrosamine and salted fish	Smoking is not a risk factor	Smoking, salty food, exposure to wood dust and iron filings.
**EBV Latency**	Type II latency	Postulated to be type II latency	Type I latency
**Key Molecular and Immune Characteristics**	NF-кB pathway activation, mutations in CDKN2A, CCND1, TP53, JAK/STAT, PI3K-Akt pathway, and Chromosomal instability phenotype EBV-CIMP (CpG island methylator phenotype). Upregulation of SSTR2.	NF-кB pathway activation, mutations in CDKN2A, CCND1, TP53, JAK/STAT. Upregulation of SSTR2.	Mutations in PIK3CA and mutations in CDKN2A. EBV-CIMP
**Somatic Mutations**	Loss of chromosomes in 3p, 9p (9p21), 11q (11q13), and 14q Hypermethylation at 6p21.3	9p21.3 deletion and 11q13.3 amplification	Hypermethylation at 6p21.3 Methylation of TSG (APC, PTEN and RASSF1A), cell adhesion molecules (THBS1 and E-cadherin), CDKN2A, MLH1, CXXC4, TIMP2 and PLXND1. Mutations in ARID1A and BCOR

EBV infection is the common oncogenic pathogen underlying several seemingly anatomically unrelated cancers, in particular, NPC, EBVaGC and PPLELC. Even though EBV is spread through droplets, it is often absent in nasopharyngeal epithelium even though EBV titres are high (48). This led to hypotheses that EBV could remain latent in other anatomical sites (48). This postulation is bolstered by the suggestion that one of the variants of NPC originates from the basal cells of the respiratory epithelium despite NPC’s squamous origin (286).

Although EBV is involved in NPC, EBVaGC and PPLELC, it enters different latency programs as evidenced by their distinct molecular profiles. EBVaGCs express LMP2A, BARTs, miR-BARTs, EBNA1, and EBER (57, 58), however, they express significantly lower levels of EBNA2 and LMP1 compared to NPC. This suggests that EBVaGC exhibits a type I latency program (287), and is more similar to burkitt’s lymphoma and distinct from NPC (30, 287, 288). On the other hand, EBV latent genes expressed in NPC include EBNA1, LMP1, LMP2, EBER, BARF, BARTS and miR-BARTs, characteristic of a type II latency program. While the type of EBV latency exhibited in LELC still remains to be uncovered, expressions of LMP1 and LMP2 strongly suggest a Type II or III EBV latency program, akin to that of NPC (122). Understanding the pathogenesis of NPC may guide current understanding of PPLELC pathogenesis.

Due to the rarity of PPLELC, there is a paucity of studies examining the role of EBV in PPLELC and our understanding is still in its infancy. Histological similarities between NPC and PPLELC suggest a closely related pathogenesis, and current perspectives of the role of EBV in NPC pathogenesis can potentially guide our future understanding of EBV in PPLELC and. Despite different cell lines of origin in NPC and PPLELC, the mutational landscape in PPLELC implies similar driver mutations in NF-κB, CDKN2A, JAK/STAT pathway. They also have similar p53 and PD-L1 regulatory patterns. Its expression of latent phase gene products such as LMP1 and LMP2 also suggests that it has a type II latency program similar to NPC. The parallels in genomic and molecular profiles of EBV positive NPC and PPLELC raise the possibility for common therapeutic strategies for advanced NPC and PPLELC. With its distribution mainly in Asia as well as its good prognosis, future studies investigating the mechanisms driving the PPLELC can potentially guide therapeutic targets. Given the histopathological and molecular similarities between NPC and PPLELC, it is unsurprising that therapeutics proven effective for NPC have demonstrated efficacy in treating PPLELC as well. This convergence in treatment strategies particularly between NPC and PPLELC could potentially extend to other EBV-related epithelial cancers in future. The development of a universal EBV-directed therapeutic approach in future holds promise to effectively treat a wide range of EBV-positive epithelial cancers, including but not limited to NPC and PPLELC. However, because of the limited studies in PPLELC, studies on EBV CTL or other novel EBV related therapies on PPLELC can help guide our understanding in this area.

The complex interplay between genetic mutations with local inflammation of the nasopharyngeal epithelium is thought to establish latent EBV infection, which is the primary driver of NPC pathogenesis (289). Despite the molecular similarities between NPC and PPLELC, more studies are required to better understand the distinct *in vivo* microenvironments of their respective host stromal components. The role of the *in vivo* microenvironment in pathogenesis is reinforced by the challenges in replicating the microenvironment *in vitro* when creating NPC cell lines, as well as the continuous loss of EBV episomes in NPC cells *in vitro (* 289). Our group is currently investigating the spatial transcriptomic differences between NPC and PPLELC. A clearer understanding of the evolutionary advantages occurring *in vivo* in both NPC and PPLELC pathogenesis can elucidate fresh avenues for therapeutic intervention.

## 10 Conclusion

Though the role of EBV in the pathogenesis of NPC is well-described in existing literature, there is still much to be understood about the role of EBV in the pathogenesis of LELC. In this review, we summarized the genetic and environmental factors that influence susceptibility to NPC, and the role of EBV in the stepwise pathogenesis of NPC. We also summarized the differences and similarities of the roles of EBV in both NPC, EBVaGC, and postulated its role in LELC pathogenesis. With increasing understanding of the role of EBV in LELC pathogenesis, better therapeutics can be developed, and novel more specific drug targets can be elucidated to improve the treatment options of EBV driven cancers.

## Author contributions

YL and CL contributed equally to manuscript writing and share first authorship. AC contributed to manuscript writing. DP contributed to the illustration of the manuscript. SH and HT both contributed to conception, guidance, and manuscript writing. All authors contributed to the article and approved the submitted version.

## References

[1] Lieberman PM. Virology. Epstein-Barr virus turns 50. Science (2014) 343:1323–5. doi: 10.1126/science.1252786 PMC4581426 24653027

[2] Ning S. Innate immune modulation in EBV infection. Herpesviridae (2011) 2:1. doi: 10.1186/2042-4280-2-1 21429244 PMC3063194

[3] Han S Tay JK Loh CJL Chu AJM Yeong JPS Lim CM. Epstein–Barr virus epithelial cancers—A comprehensive understanding to drive novel therapies. Front Immunol (2021) 12:734293. doi: 10.3389/fimmu.2021.734293 34956172 PMC8702733

[4] Chan ATC Teo PML Huang DP. Pathogenesis and treatment of nasopharyngeal carcinoma. Semin Oncol (2004) 31:794–801. doi: 10.1053/j.seminoncol.2004.09.008 15599857

[5] Boussen H Ghorbal L Naouel L Bouaouina N Gritli S Benna F. Nasopharyngeal cancer (NPC) around the Mediterranean area: standard of care. Crit Rev Oncol Hematol (2012) 84(Suppl 1):e106–9. doi: 10.1016/j.critrevonc.2010.09.005 21177119

[6] Niedobitek G. Epstein-Barr virus infection in the pathogenesis of nasopharyngeal carcinoma. Mol Pathol (2000) 53:248–54. doi: 10.1136/mp.53.5.248 PMC1186977 11091848

[7] Eales L-J. Epstein-Barr virus and human disease. (Springer) Vaccine (1990) 8(1):95. doi:10.1016/0264-410x(90)90202-w

[8] Yu MC Yuan J-M. Epidemiology of nasopharyngeal carcinoma. Semin Cancer Biol (2002) 12:421–9. doi: 10.1016/S1044579X02000858 12450728

[9] Tay JK Siow CH Goh HL Lim CM Hsu PP Chan SH. A comparison of EBV serology and serum cell-free DNA as screening tools for nasopharyngeal cancer: Results of the Singapore NPC screening cohort. Int J Cancer (2020) 146:2923–31. doi: 10.1002/ijc.32774 31705522

[10] Tao Q Chan ATC. Nasopharyngeal carcinoma: molecular pathogenesis and therapeutic developments. Expert Rev Mol Med (2007) 9:1–24. doi: 10.1017/S1462399407000312 17477889

[11] Bray F Colombet M Mery L Piñeros M Znaor A Zanetti R. Cancer incidence in five continents, Vol. XI Vol. 2017. . Lyon: International Agency for Research on Cancer (2018).

[12] Chang ET Ye W Zeng Y-X Adami H-O. The evolving epidemiology of nasopharyngeal carcinoma. Cancer Epidemiol Biomarkers Prev (2021) 30:1035–47. doi: 10.1158/1055-9965.EPI-20-1702 33849968

[13] Pathmanathan R Prasad U Sadler R Flynn K Raab-Traub N. Clonal proliferations of cells infected with Epstein–Barr virus in preinvasive lesions related to nasopharyngeal carcinoma. N Engl J Med (1995) 333:693–8. doi: 10.1056/NEJM199509143331103 7637746

[14] Coghill AE Hsu W-L Pfeiffer RM Juwana H Yu KJ Lou P-J. Epstein–Barr virus serology as a potential screening marker for nasopharyngeal carcinoma among high-risk individuals from multiplex families in TaiwanEBV serology and NPC screening in high-risk families. Cancer Epidemiol Biomarkers Prev (2014) 23:1213–9. doi: 10.1158/1055-9965.EPI-13-1262 PMC4082438 24769890

[15] Ji MF Wang DK Yu YL Guo YQ Liang JS Cheng WM. Sustained elevation of Epstein–Barr virus antibody levels preceding clinical onset of nasopharyngeal carcinoma. Br J Cancer (2007) 96:623–30. doi: 10.1038/sj.bjc.6603609 PMC2360049 17285127

[16] Brennan B. Nasopharyngeal carcinoma. Orphanet J Rare Dis (2006) 1:23. doi: 10.1186/1750-1172-1-23 16800883 PMC1559589

[17] Adham M Kurniawan AN Muhtadi AI Roezin A Hermani B Gondhowiardjo S. Nasopharyngeal carcinoma in Indonesia: epidemiology, incidence, signs, and symptoms at presentation. Chin J Cancer (2012) 31:185–96. doi: 10.5732/cjc.011.10328 PMC3777476 22313595

[18] Byrd DR Brookland RK Washington MK Gershenwald JE Compton CC Hess KR. AJCC Cancer Staging Manual. (Vol. 1024). In Amin MB. Edge SB. Greene FL. eds. (New York: Springer International Publishing) (2017) 17 p.

[19] Camargo MC Kim K-M Matsuo K Torres J Liao LM Morgan DR. Anti-Helicobacter pylori Antibody Profiles in Epstein-Barr virus (EBV)-Positive and EBV-Negative Gastric Cancer. Helicobacter (2016) 21:153–7. doi: 10.1111/hel.12249 PMC5003173 26251258

[20] Shinozaki-Ushiku A Kunita A Fukayama M. Update on Epstein-Barr virus and gastric cancer (review). Int J Oncol (2015) 46:1421–34. doi: 10.3892/ijo.2015.2856 25633561

[21] Lee J-H Kim S-H Han S-H An J-S Lee E-S Kim Y-S. Clinicopathological and molecular characteristics of Epstein-Barr virus-associated gastric carcinoma: a meta-analysis. J Gastroenterol Hepatol (2009) 24:354–65. doi: 10.1111/j.1440-1746.2009.05775.x 19335785

[22] Naseem M Barzi A Brezden-Masley C Puccini A Berger MD Tokunaga R. Outlooks on Epstein-Barr virus associated gastric cancer. Cancer Treat Rev (2018) 66:15–22. doi: 10.1016/j.ctrv.2018.03.006 29631196 PMC5964025

[23] Koriyama C Akiba S Minakami Y Eizuru Y. Environmental factors related to Epstein-Barr virus-associated gastric cancer in Japan. J Exp Clin Cancer Res (2005) 24:547–53. 16471317

[24] Kaizaki Y Hosokawa O Sakurai S Fukayama M. Epstein-Barr virus-associated gastric carcinoma in the remnant stomach: *de novo* and metachronous gastric remnant carcinoma. J Gastroenterol (2005) 40:570–7. doi: 10.1007/s00535-005-1590-3 16007390

[25] Camargo MC Koriyama C Matsuo K Kim W-H Herrera-Goepfert R Liao LM. Case-case comparison of smoking and alcohol risk associations with Epstein-Barr virus-positive gastric cancer. Int J Cancer (2014) 134:948–53. doi: 10.1002/ijc.28402 PMC3961829 23904115

[26] Chuang M-K Hong R-L. Nasopharyngeal carcinoma and pulmonary lymphoepithelioma-like carcinoma – metastases or synchronous second primary cancer. J Cancer Surviv (2015) 2:248–54. doi: 10.6323/JCRP.2015.2.3.08

[27] Ho JC Wong MP Lam WK. Lymphoepithelioma-like carcinoma of the lung. Respirology (2006) 11:539–45. doi: 10.1111/j.1440-1843.2006.00910.x 16916325

[28] Bégin LR Eskandari J Joncas J Panasci L. Epstein-Barr virus related lymphoepithelioma-like carcinoma of lung. J Surg Oncol (1987) 36:280–3. doi: 10.1002/jso.2930360413 2826922

[29] Zhao W Deng N Gao X Chen T-B Xie J Li Q. Primary lymphoepithelioma-like carcinoma of salivary glands: a clinicopathological study of 21 cases. Int J Clin Exp Pathol (2014) 7:7951–6. PMC4270609 25550837

[30] Cheng N Hui D-Y Liu Y Zhang N-N Jiang Y Han J. Is gastric lymphoepithelioma-like carcinoma a special subtype of EBV-associated gastric carcinoma? New insight based on clinicopathological features and EBV genome polymorphisms. Gastric Cancer (2015) 18:246–55. doi: 10.1007/s10120-014-0376-9 24771002

[31] Shibata D Tokunaga M Uemura Y Sato E Tanaka S Weiss LM. Association of Epstein-Barr virus with undifferentiated gastric carcinomas with intense lymphoid infiltration. Lymphoepithelioma-like carcinoma. Am J Pathol (1991) 139:469–74. PMC1886210 1653517

[32] Ose N Kawagishi S Funaki S Kanou T Fukui E Kimura K. Thymic lymphoepithelial carcinoma associated with Epstein-Barr virus: experiences and literature review. Cancers (2021) 13(19):4794. doi: 10.3390/cancers13194794 34638279 PMC8507618

[33] Mori Y Akagi K Yano M Sashiyama H Tsutsumi O Hamahata Y. Lymphoepithelioma-like carcinoma of the colon. Case Rep Gastroenterol (2013) 7:127–33. doi: 10.1159/000348765 PMC3617975 23626513

[34] Archwamety A Ruangchira-Urai R Akewanlop C Korphaisarn K. Primary pulmonary lymphoepithelioma-like carcinoma treated with immunotherapy: A case report and literature review. Thorac Cancer (2022) 13:2539–41. doi: 10.1111/1759-7714.14580 PMC9436678 35830974

[35] Khandakar B Liu J-R Thung S Li Y Rhee H Kagen AC. Lymphoepithelioma-like neoplasm of the biliary tract with “probable low Malignant potential”. Histopathology (2022) 80:720–8. doi: 10.1111/his.14580 34608670

[36] Jiang W-Y Wang R Pan X-F Shen Y-Z Chen T-X Yang Y-H. Clinicopathological features and prognosis of primary pulmonary lymphoepithelioma-like carcinoma. J Thorac Dis (2016) 8:2610–6. doi: 10.21037/jtd.2016.08.40 PMC5059351 27747015

[37] Huang C-J Chan K-Y Lee M-Y Hsu L-H Chu N-M Feng A-C. Computed tomography characteristics of primary pulmonary lymphoepithelioma-like carcinoma. Br J Radiol (2007) 80:803–6. doi: 10.1259/bjr/27788443 17875600

[38] Sun Y-H Lin S-W Hsieh C-C Yeh Y-C Tu C-C Chen K-J. Treatment outcomes of patients with different subtypes of large cell carcinoma of the lung. Ann Thorac Surg (2014) 98:1013–9. doi: 10.1016/j.athoracsur.2014.05.012 25085555

[39] Curcio LD Cohen JS Grannis FW Jr Paz IB Chilcote R Weiss LM. Primary lymphoepithelioma-like carcinoma of the lung in a child. Report of an Epstein-Barr virus-related neoplasm. Chest (1997) 111:250–1. doi: 10.1378/chest.111.1.250 8996028

[40] Castro CY Ostrowski ML Barrios R Green LK Popper HH Powell S. Relationship between Epstein-Barr virus and lymphoepithelioma-like carcinoma of the lung: a clinicopathologic study of 6 cases and review of the literature. Hum Pathol (2001) 32:863–72. doi: 10.1053/hupa.2001.26457 11521232

[41] Grimes BS Albores J Barjaktarevic I. A 65-year-old man with persistent cough and large nodular opacity. Chest (2015) 147:e13–7. doi: 10.1378/chest.14-1172 25560867

[42] He J Shen J Pan H Huang J Liang W He J. Pulmonary lymphoepithelioma-like carcinoma: a Surveillance, Epidemiology, and End Results database analysis. J Thorac Dis (2015) 7:2330–8. doi: 10.3978/j.issn.2072-1439.2015.12.62 PMC4703678 26793355

[43] Fan Y Li C Qin J Lu H. Primary pulmonary lymphoepithelioma-like carcinoma. Med Oncol (2020) 37:20. doi: 10.1007/s12032-020-1344-3 32146584

[44] Ye L Luo D Li H Zheng H He S Lin L. A clinical analysis and literature review of six cases with primary pulmonary lymphoepithelioma-like carcinoma. Comput Math Methods Med (2022) 2022:1086697. doi: 10.1155/2022/1086697 35529269 PMC9076294

[45] Zhang L Hao T Wei Y Dong M Xiong Y. Primary pulmonary lymphoepithelioma-like carcinoma: A case report of pathological complete response (pCR) by neoadjuvant treatment. Medicine (2021) 100:e24987. doi: 10.1097/MD.0000000000024987 33725970 PMC7982184

[46] Raghupathy R Hui EP Chan ATC. Epstein-Barr virus as a paradigm in nasopharyngeal cancer: from lab to clinic. Am Soc Clin Oncol Educ Book (2014) 34(1):149–53. doi: 10.14694/EdBook_AM.2014.34.149 24857071

[47] Hau PM Lung HL Wu M Tsang CM Wong K-L Mak NK. Targeting Epstein-Barr virus in nasopharyngeal carcinoma. Front Oncol (2020) 10:600. doi: 10.3389/fonc.2020.00600 32528868 PMC7247807

[48] Poh SS Chua MLK Wee JTS. Carcinogenesis of nasopharyngeal carcinoma: an alternate hypothetical mechanism. Chin J Cancer (2016) 35:9. doi: 10.1186/s40880-015-0068-9 26738743 PMC4704291

[49] Salano VE Mwakigonja AR Abdulshakoor A Kahinga AA Richard EM. Epstein-Barr virus latent membrane protein-1 expression in nasopharyngeal carcinoma. JCO Glob Oncol (2021) 7:1406–12. doi: 10.1200/GO.21.00120 PMC8457857 34546798

[50] Houldcroft CJ Kellam P. Host genetics of Epstein-Barr virus infection, latency and disease. Rev Med Virol (2015) 25:71–84. doi: 10.1002/rmv.1816 25430668 PMC4407908

[51] Kliszczewska E Jarzyński A Boguszewska A Pasternak J Polz-Dacewicz M. Epstein-Barr Virus – pathogenesis, latency and cancers. J Pre-Clinical Clin Res (2017) 11:142–6. doi: 10.26444/jpccr/81214

[52] Tsao SW Tsang CM Pang PS Zhang G Chen H Lo KW. The biology of EBV infection in human epithelial cells. Semin Cancer Biol (2012) 22:137–43. doi: 10.1016/j.semcancer.2012.02.004 22497025

[53] Rickinson AB. Co-infections, inflammation and oncogenesis: future directions for EBV research. Semin Cancer Biol (2014) 26:99–115. doi: 10.1016/j.semcancer.2014.04.004 24751797

[54] Zhang H Li Y Wang H-B Zhang A Chen M-L Fang Z-X. Ephrin receptor A2 is an epithelial cell receptor for Epstein-Barr virus entry. Nat Microbiol (2018) 3:1–8. doi: 10.1038/s41564-017-0080-8 29292383

[55] Hayashi K Teramoto N Akagi T Sasaki Y Suzuki T. *In situ* detection of Epstein-Barr virus in the gastric glands with intestinal metaplasia. Am J Gastroenterol (1996) 91:1481. doi: 10.3389/fendo.2021.767314 8678040

[56] Fukayama M Hayashi Y Iwasaki Y Chong J Ooba T Takizawa T. Epstein-Barr virus-associated gastric carcinoma and Epstein-Barr virus infection of the stomach. Lab Invest (1994) 71:73–81. 8041121

[57] Sugiura M Imai S Tokunaga M Koizumi S Uchizawa M Okamoto K. Transcriptional analysis of Epstein-Barr virus gene expression in EBV-positive gastric carcinoma: unique viral latency in the tumour cells. Br J Cancer (1996) 74:625–31. doi: 10.1038/bjc.1996.412 PMC2074674 8761381

[58] Luo B Wang Y Wang X-F Liang H Yan L-P Huang B-H. Expression of Epstein-Barr virus genes in EBV-associated gastric carcinomas. World J Gastroenterol (2005) 11:629–33. doi: 10.3748/wjg.v11.i5.629 PMC4250728 15655811

[59] Iwakiri D Eizuru Y Tokunaga M Takada K. Autocrine growth of Epstein-Barr virus-positive gastric carcinoma cells mediated by an Epstein-Barr virus-encoded small RNA. Cancer Res (2003) 63:7062–7. 14612496

[60] Banerjee AS Pal AD Banerjee S. Epstein-Barr virus-encoded small non-coding RNAs induce cancer cell chemoresistance and migration. Virology (2013) 443:294–305. doi: 10.1016/j.virol.2013.05.020 23791019

[61] Sivachandran N Dawson CW Young LS Liu F-F Middeldorp J Frappier L. Contributions of the Epstein-Barr virus EBNA1 protein to gastric carcinoma. J Virol (2012) 86:60–8. doi: 10.1128/JVI.05623-11 PMC3255905 22013060

[62] Kim S-M Hur DY Hong S-W Kim JH. EBV-encoded EBNA1 regulates cell viability by modulating miR34a-NOX2-ROS signaling in gastric cancer cells. Biochem Biophys Res Commun (2017) 494:550–5. doi: 10.1016/j.bbrc.2017.10.095 29061308

[63] Wang J Liu W Zhang X Zhang Y Xiao H Luo B. LMP2A induces DNA methylation and expression repression of AQP3 in EBV-associated gastric carcinoma. Virology (2019) 534:87–95. doi: 10.1016/j.virol.2019.06.006 31220652

[64] Xu Q Du J Liu B. Lymphoepithelioma-like gastric carcinoma located in the lesser curvature of the gastric body: A case report and review of the literature. Mol Clin Oncol (2016) 4:405–8. doi: 10.3892/mco.2015.717 PMC4774426 26998292

[65] Ding Y Sun Z You W Zhang S Chang C Yan S. Lymphoepithelioma-like intrahepatic cholangiocarcinoma with Epstein-Barr virus infection: report of a rare case. Ann Transl Med (2019) 7:497. doi: 10.21037/atm.2019.08.105 31700933 PMC6803244

[66] Xie C Xu X Wu B Yang K-Y Huang J. Primary pulmonary lymphoepithelioma-like carcinoma in non-endemic region: A case report and literature review. Medicine (2018) 97:e9976. doi: 10.1097/MD.0000000000009976 29465599 PMC5841981

[67] Ngan RKC Yip TTC Cheng W-W Chan JKC Cho WCS Ma VWS. Circulating Epstein-Barr virus DNA in serum of patients with lymphoepithelioma-like carcinoma of the lung: a potential surrogate marker for monitoring disease. Clin Cancer Res (2002) 8:986–94. 11948104

[68] Xie M Wu X Wang F Zhang J Ben X Zhang J. Clinical significance of plasma Epstein-Barr virus DNA in pulmonary lymphoepithelioma-like carcinoma (LELC) patients. J Thorac Oncol (2018) 13:218–27. doi: 10.1016/j.jtho.2017.10.031 29191777

[69] Jia W-H Qin H-D. Non-viral environmental risk factors for nasopharyngeal carcinoma: a systematic review. Semin Cancer Biol (2012) 22:117–26. doi: 10.1016/j.semcancer.2012.01.009 22311401

[70] Putera I Ramadhan MG Anindya S Sutanto NR Kurniawan A Hosea FN. Relationship between salted fish consumption and nasopharyngeal carcinoma: an evidence-based case report. Acta Med Indones (2015) 47:72–7. 25948772

[71] Armstrong RW Imrey PB Lye MS Armstrong MJ Yu MC Sani S. Nasopharyngeal carcinoma in Malaysian Chinese: occupational exposures to particles, formaldehyde and heat. Int J Epidemiol (2000) 29:991–8. doi: 10.1093/ije/29.6.991 11101539

[72] Armstrong RW Armstrong MJ Yu MC Henderson BE. Salted fish and inhalants as risk factors for nasopharyngeal carcinoma in Malaysian Chinese. Cancer Res (1983) 43:2967–70. 6850606

[73] Yang XR Diehl S Pfeiffer R Chen C-J Hsu W-L Dosemeci M. Evaluation of risk factors for nasopharyngeal carcinoma in high-risk nasopharyngeal carcinoma families in Taiwan. Cancer Epidemiol Biomarkers Prev (2005) 14:900–5. doi: 10.1158/1055-9965.EPI-04-0680 15826929

[74] Vaughan TL Stewart PA Teschke K Lynch CF Swanson GM Lyon JL. Occupational exposure to formaldehyde and wood dust and nasopharyngeal carcinoma. Occup Environ Med (2000) 57:376–84. doi: 10.1136/oem.57.6.376 PMC1739963 10810126

[75] Hildesheim A Dosemeci M Chan CC Chen CJ Cheng YJ Hsu MM. Occupational exposure to wood, formaldehyde, and solvents and risk of nasopharyngeal carcinoma. Cancer Epidemiol Biomarkers Prev (2001) 10:1145–53. 11700262

[76] Hardell L Johansson B Axelson O. Epidemiological study of nasal and nasopharyngeal cancer and their relation to phenoxy acid or chlorophenol exposure. Am J Ind Med (1982) 3:247–57. doi: 10.1002/ajim.4700030304 6303119

[77] Yu MC Garabrant DH Huang TB Henderson BE. Occupational and other non-dietary risk factors for nasopharyngeal carcinoma in Guangzhou, China. Int J Cancer (1990) 45:1033–9. doi: 10.1002/ijc.2910450609 2351484

[78] Henderson BE Louie E SooHoo Jing J Buell P Gardner MB. Risk factors associated with nasopharyngeal carcinoma. N Engl J Med (1976) 295:1101–6. doi: 10.1056/NEJM197611112952003 980005

[79] Tay CK Chua YC Takano A Min Chee MY Lim W-T Lim C. Primary pulmonary lymphoepithelioma-like carcinoma in Singapore. Ann Thorac Med (2018) 13:30–5. doi: 10.4103/atm.ATM_304_17 PMC5772105 29387253

[80] Li X Fasano R Wang E Yao K-T Marincola FM. HLA associations with nasopharyngeal carcinoma. Curr Mol Med (2009) 9:751–65. doi: 10.2174/156652409788970698 PMC3407690 19689302

[81] Tsao SW Tsang CM Lo KW. Epstein-Barr virus infection and nasopharyngeal carcinoma. Philos Trans R Soc Lond B Biol Sci (2017) 372(1732):20160270. doi: 10.1098/rstb.2016.0270 28893937 PMC5597737

[82] Zhou X-X Jia W-H Shen G-P Qin H Yu X-J Chen L-Z. Sequence variants in toll-like receptor 10 are associated with nasopharyngeal carcinoma risk. Cancer Epidemiol Biomarkers Prev (2006) 15:862–6. doi: 10.1158/1055-9965.EPI-05-0874 16702361

[83] Song C Chen L-Z Zhang R-H Yu X-J Zeng Y-X. Functional variant in the 3’-untranslated region of toll-like receptor 4 is associated with nasopharyngeal carcinoma risk. Cancer Biol Ther (2006) 5:1285–91. doi: 10.4161/cbt.5.10.3304 16969132

[84] He J-F Jia W-H Fan Q Zhou X-X Qin H-D Shugart YY. Genetic polymorphisms of TLR3 are associated with Nasopharyngeal carcinoma risk in Cantonese population. BMC Cancer (2007) 7:194. doi: 10.1186/1471-2407-7-194 17939877 PMC2121103

[85] Dai Q Li XP Chai L Long HA Yang ZH. Polymorphisms of Toll-like receptor 9 are associated with nasopharyngeal carcinoma susceptibility. Tumour Biol (2014) 35:3247–53. doi: 10.1007/s13277-013-1424-5 24504675

[86] Jabłońska A Studzińska M Szenborn L Wiśniewska-Ligier M Karlikowska-Skwarnik M Gęsicki T. TLR4 896A/G and TLR9 1174G/A polymorphisms are associated with the risk of infectious mononucleosis. Sci Rep (2020) 10:13154. doi: 10.1038/s41598-020-70129-4 32753695 PMC7403730

[87] Kutikhin AG. Impact of Toll-like receptor 4 polymorphisms on risk of cancer. Hum Immunol (2011) 72:193–206. doi: 10.1016/j.humimm.2010.11.003 21081146

[88] Liu S Wang X Shi Y Han L Zhao Z Zhao C. Toll-like receptor gene polymorphisms and susceptibility to Epstein-Barr virus-associated and -negative gastric carcinoma in Northern China. Saudi J Gastroenterol (2015) 21:95–103. doi: 10.4103/1319-3767.153832 25843196 PMC4392582

[89] Dworzanska A Strycharz-Dudziak M Dworzanski J Stec A Rajtar B Drop B. The role of toll-like receptor 9 (TLR9) in Epstein-Barr virus-associated gastric cancer. Curr Issues Pharm Med Sci (2020) 33:106–11. doi: 10.2478/cipms-2020-0020

[90] Allday MJ Crawford DH. Role of epithelium in EBV persistence and pathogenesis of B-cell tumours. Lancet (1988) 1:855–7. doi: 10.1016/S0140-6736(88)91604-2 2895366

[91] Young LS Dawson CW. Epstein-Barr virus and nasopharyngeal carcinoma. Chin J Cancer (2014) 33:581–90. doi: 10.5732/cjc.014.10197 PMC4308653 25418193

[92] Lo KW Teo PM Hui AB To KF Tsang YS Chan SY. High resolution allelotype of microdissected primary nasopharyngeal carcinoma. Cancer Res (2000) 60:3348–53. 10910036

[93] Chan AS To KF Lo KW Mak KF Pak W Chiu B. High frequency of chromosome 3p deletion in histologically normal nasopharyngeal epithelia from southern Chinese. Cancer Res (2000) 60:5365–70. 11034072

[94] Tsang CM Lui VWY Bruce JP Pugh TJ Lo KW. Translational genomics of nasopharyngeal cancer. Semin Cancer Biol (2020) 61:84–100. doi: 10.1016/j.semcancer.2019.09.006 31521748

[95] Dai W Cheung AKL Ko JMY Cheng Y Zheng H Ngan RKC. Comparative methylome analysis in solid tumors reveals aberrant methylation at chromosome 6p in nasopharyngeal carcinoma. Cancer Med (2015) 4:1079–90. doi: 10.1002/cam4.451 PMC4529346 25924914

[96] Niller HH Banati F Salamon D Minarovits J. Epigenetic alterations in Epstein-Barr virus-associated diseases. Adv Exp Med Biol (2016) 879:39–69. doi: 10.1007/978-3-319-24738-0_3 26659263

[97] Or YY-Y Hui AB-Y To K-F Lam CN-Y Lo K-W. PIK3CA mutations in nasopharyngeal carcinoma. Int J Cancer (2006) 118:1065–7. doi: 10.1002/ijc.21444 16114017

[98] Hui AB-Y Or YY-Y Takano H Tsang RK-Y To K-F Guan X-Y. Array-based comparative genomic hybridization analysis identified cyclin D1 as a target oncogene at 11q13.3 in nasopharyngeal carcinoma. Cancer Res (2005) 65:8125–33. doi: 10.1158/0008-5472.CAN-05-0648 16166286

[99] Or YY-Y Chung GT-Y To K-F Chow C Choy K-W Tong CY-K. Identification of a novel 12p13.3 amplicon in nasopharyngeal carcinoma. J Pathol (2010) 220:97–107. doi: 10.1002/path.2609 19718711

[100] Li L Zhang Y Fan Y Sun K Su X Du Z. Characterization of the nasopharyngeal carcinoma methylome identifies aberrant disruption of key signaling pathways and methylated tumor suppressor genes. Epigenomics (2015) 7:155–73. doi: 10.2217/epi.14.79 25479246

[101] Zhao W Mo Y Wang S Midorikawa K Ma N Hiraku Y. Quantitation of DNA methylation in Epstein-Barr virus–associated nasopharyngeal carcinoma by bisulfite amplicon sequencing. BMC Cancer (2017) 17:489. doi: 10.1186/s12885-017-3482-3 28716111 PMC5514474

[102] Tsai C-L Li H-P Lu Y-J Hsueh C Liang Y Chen C-L. Activation of DNA methyltransferase 1 by EBV LMP1 Involves c-Jun NH(2)-terminal kinase signaling. Cancer Res (2006) 66:11668–76. doi: 10.1158/0008-5472.CAN-06-2194 17178861

[103] Tsai C-N Tsai C-L Tse K-P Chang H-Y Chang Y-S. The Epstein-Barr virus oncogene product, latent membrane protein 1, induces the downregulation of E-cadherin gene expression via activation of DNA methyltransferases. Proc Natl Acad Sci U.S.A. (2002) 99:10084–9. doi: 10.1073/pnas.152059399 PMC126628 12110730

[104] Hino R Uozaki H Murakami N Ushiku T Shinozaki A Ishikawa S. Activation of DNA methyltransferase 1 by EBV latent membrane protein 2A leads to promoter hypermethylation of PTEN gene in gastric carcinoma. Cancer Res (2009) 69:2766–74. doi: 10.1158/0008-5472.CAN-08-3070 19339266

[105] Matsusaka K Funata S Fukuyo M Seto Y Aburatani H Fukayama M. Epstein-Barr virus infection induces genome-wide *de novo* DNA methylation in non-neoplastic gastric epithelial cells. J Pathol (2017) 242:391–9. doi: 10.1002/path.4909 28418084

[106] Jiang W Liu N Chen X-Z Sun Y Li B Ren X-Y. Genome-wide identification of a methylation gene panel as a prognostic biomarker in nasopharyngeal carcinoma. Mol Cancer Ther (2015) 14:2864–73. doi: 10.1158/1535-7163.MCT-15-0260 26443805

[107] Zhang L Wang R Xie Z. The roles of DNA methylation on the promotor of the Epstein–Barr virus (EBV) gene and the genome in patients with EBV-associated diseases. Appl Microbiol Biotechnol (2022) 106:4413–26. doi: 10.1007/s00253-022-12029-3 PMC9259528 35763069

[108] Zhang Z Sun D Van DN Tang A Hu L Huang G. Inactivation of RASSF2A by promoter methylation correlates with lymph node metastasis in nasopharyngeal carcinoma. Int J Cancer (2007) 120:32–8. doi: 10.1002/ijc.22185 17013896

[109] Zhou X Xiao X Huang T Du C Wang S Mo Y. Epigenetic inactivation of follistatin-like 1 mediates tumor immune evasion in nasopharyngeal carcinoma. Oncotarget (2016) 7:16433–44. doi: 10.18632/oncotarget.7654 PMC4941326 26918942

[110] Cheng Y Stanbridge EJ Kong H Bengtsson U Lerman MI Lung ML. A functional investigation of tumor suppressor gene activities in a nasopharyngeal carcinoma cell line HONE1 using a monochromosome transfer approach. Genes Chromosomes Cancer (2000) 28:82–91. doi: 10.1002/(SICI)1098-2264(200005)28:1<82::AID-GCC10>3.0.CO;2-8 10738306

[111] Lo KW Cheung ST Leung SF van Hasselt A Tsang YS Mak KF. Hypermethylation of the p16 gene in nasopharyngeal carcinoma. Cancer Res (1996) 56:2721–5. 8665502

[112] Li J Gong P Lyu X Yao K Li X Peng H. Aberrant CpG island methylation of PTEN is an early event in nasopharyngeal carcinoma and a potential diagnostic biomarker. Oncol Rep (2014) 31:2206–12. doi: 10.3892/or.2014.3061 24604064

[113] Tsao SW Liu Y Wang X Yuen PW Leung SY Yuen ST. The association of E-cadherin expression and the methylation status of the E-cadherin gene in nasopharyngeal carcinoma cells. Eur J Cancer (2003) 39:524–31. doi: 10.1016/S0959-8049(02)00494-X 12751385

[114] Leong MML Lung ML. The impact of Epstein-Barr virus infection on epigenetic regulation of host cell gene expression in epithelial and lymphocytic Malignancies. Front Oncol (2021) 11:629780. doi: 10.3389/fonc.2021.629780 33718209 PMC7947917

[115] Yau TO Tang C-M Yu J. Epigenetic dysregulation in Epstein-Barr virus-associated gastric carcinoma: disease and treatments. World J Gastroenterol (2014) 20:6448–56. doi: 10.3748/wjg.v20.i21.6448 PMC4047330 24914366

[116] Kang GH Lee S Kim WH Lee HW Kim JC Rhyu M-G. Epstein-barr virus-positive gastric carcinoma demonstrates frequent aberrant methylation of multiple genes and constitutes CpG island methylator phenotype-positive gastric carcinoma. Am J Pathol (2002) 160:787–94. doi: 10.1016/S0002-9440(10)64901-2 PMC1867170 11891177

[117] Vo QN Geradts J Gulley ML Boudreau DA Bravo JC Schneider BG. Epstein-Barr virus in gastric adenocarcinomas: association with ethnicity and CDKN2A promoter methylation. J Clin Pathol (2002) 55:669–75. doi: 10.1136/jcp.55.9.669 PMC1769746 12194996

[118] Chong J-M Sakuma K Sudo M Ushiku T Uozaki H Shibahara J. Global and non-random CpG-island methylation in gastric carcinoma associated with Epstein-Barr virus. Cancer Sci (2003) 94:76–80. doi: 10.1111/j.1349-7006.2003.tb01355.x 12708478 PMC11160188

[119] Sudo M Chong J-M Sakuma K Ushiku T Uozaki H Nagai H. Promoter hypermethylation of E-cadherin and its abnormal expression in Epstein-Barr virus-associated gastric carcinoma. Int J Cancer (2004) 109:194–9. doi: 10.1002/ijc.11701 14750169

[120] Han AJ Xiong M Zong YS. Association of Epstein-Barr virus with lymphoepithelioma-like carcinoma of the lung in southern China. Am J Clin Pathol (2000) 114:220–6. doi: 10.1309/148K-ND54-6NJX-NA61 10941337

[121] Dong M Chen J-N Huang J-T Gong L-P Shao C-K. The roles of EBV-encoded microRNAs in EBV-associated tumors. Crit Rev Oncol Hematol (2019) 135:30–8. doi: 10.1016/j.critrevonc.2019.01.014 30819444

[122] Chau S-L Tong JH-M Chow C Kwan JS-H Lung RW-M Chung L-Y. Distinct molecular landscape of Epstein–Barr virus associated pulmonary lymphoepithelioma-like carcinoma revealed by genomic sequencing. Cancers (2020) 12:2065. doi: 10.3390/cancers12082065 32726920 PMC7463519

[123] Hong S Liu D Luo S Fang W Zhan J Fu S. The genomic landscape of Epstein-Barr virus-associated pulmonary lymphoepithelioma-like carcinoma. Nat Commun (2019) 10:3108. doi: 10.1038/s41467-019-10902-w 31311932 PMC6635366

[124] Yi M Cai J Li J Chen S Zeng Z Peng Q. Rediscovery of NF-κB signaling in nasopharyngeal carcinoma: How genetic defects of NF-κB pathway interplay with EBV in driving oncogenesis? J Cell Physiol (2018) 233:5537–49. doi: 10.1002/jcp.26410 29266238

[125] Liu X Liu J Qiu H Kong P Chen S Li W. Prognostic significance of Epstein-Barr virus infection in gastric cancer: a meta-analysis. BMC Cancer (2015) 15:782. doi: 10.1186/s12885-015-1813-9 26498209 PMC4619309

[126] Tchelebi L Ashamalla H Graves PR. Mutant p53 and the response to chemotherapy and radiation. Subcell Biochem (2014) 85:133–59. doi: 10.1007/978-94-017-9211-0_8 25201193

[127] Liu Q Ma G Yang H Wen J Li M Yang H. Lack of epidermal growth factor receptor gene mutations in exons 19 and 21 in primary lymphoepithelioma-like carcinoma of the lung. Thorac Cancer (2014) 5:63–7. doi: 10.1111/1759-7714.12060 PMC4704286 26766974

[128] Chang AMV Chiosea SI Altman A Pagdanganan HA Ma C. Programmed death-ligand 1 expression, microsatellite instability, Epstein-Barr virus, and human papillomavirus in nasopharyngeal carcinomas of patients from the Philippines. Head Neck Pathol (2017) 11:203–11. doi: 10.1007/s12105-016-0765-y PMC5429283 27807760

[129] Dacic S Lomago D Hunt JL Sepulveda A Yousem SA. Microsatellite instability is uncommon in lymphoepithelioma-like carcinoma of the lung. Am J Clin Pathol (2007) 127:282–6. doi: 10.1309/CRCU356U7146YC31 17210524

[130] Becnel D Abdelghani R Nanbo A Avilala J Kahn J Li L. Pathogenic role of Epstein-Barr virus in lung cancers. Viruses (2021) 13(5):877. doi: 10.3390/v13050877 34064727 PMC8151745

[131] Smatti MK Al-Sadeq DW Ali NH Pintus G Abou-Saleh H Nasrallah GK. Epstein–Barr virus epidemiology, serology, and genetic variability of LMP-1 oncogene among healthy population: an update. Front Oncol (2018) 8:211. doi: 10.3389/fonc.2018.00211 29951372 PMC6008310

[132] Chesnokova LS Hutt-Fletcher LM. Fusion of Epstein-Barr virus with epithelial cells can be triggered by αvβ5 in addition to αvβ6 and αvβ8, and integrin binding triggers a conformational change in glycoproteins gHgL. J Virol (2011) 85:13214–23. doi: 10.1128/JVI.05580-11 PMC3233123 21957301

[133] Chen J Longnecker R. Epithelial cell infection by Epstein-Barr virus. FEMS Microbiol Rev (2019) 43:674–83. doi: 10.1093/femsre/fuz023 PMC7317989 31584659

[134] Chen J Sathiyamoorthy K Zhang X Schaller S Perez White BE Jardetzky TS. Ephrin receptor A2 is a functional entry receptor for Epstein-Barr virus. Nat Microbiol (2018) 3:172–80. doi: 10.1038/s41564-017-0081-7 PMC5972547 29292384

[135] Rosemarie Q Sugden B. Epstein–Barr virus: how its lytic phase contributes to oncogenesis. Microorganisms (2020) 8:1824. doi: 10.3390/microorganisms8111824 33228078 PMC7699388

[136] Lee C-H Yeh T-H Lai H-C Wu S-Y Su I-J Takada K. Epstein-Barr virus Zta-induced immunomodulators from nasopharyngeal carcinoma cells upregulate interleukin-10 production from monocytes. J Virol (2011) 85:7333–42. doi: 10.1128/JVI.00182-11 PMC3126557 21543473

[137] Phan AT Fernandez SG Somberg JJ Keck KM Miranda JL. Epstein-Barr virus latency type and spontaneous reactivation predict lytic induction levels. Biochem Biophys Res Commun (2016) 474:71–5. doi: 10.1016/j.bbrc.2016.04.070 PMC4860101 27091426

[138] Hu L Lin Z Wu Y Dong J Zhao B Cheng Y. Comprehensive profiling of EBV gene expression in nasopharyngeal carcinoma through paired-end transcriptome sequencing. Front Med (2016) 10:61–75. doi: 10.1007/s11684-016-0436-0 26969667

[139] Minamitani T Ma Y Zhou H Kida H Tsai C-Y Obana M. Mouse model of Epstein–Barr virus LMP1- and LMP2A-driven germinal center B-cell lymphoproliferative disease. Proc Natl Acad Sci (2017) 114:4751–6. doi: 10.1073/pnas.1701836114 PMC5422827 28351978

[140] Raab-Traub N. Nasopharyngeal carcinoma: an evolving role for the Epstein-Barr virus. Curr Top Microbiol Immunol (2015) 390:339–63. doi: 10.1007/978-3-319-22822-8_14 26424653

[141] Chung GT-Y Lou WP-K Chow C To K-F Choy K-W Leung AW-C. Constitutive activation of distinct NF-κB signals in EBV-associated nasopharyngeal carcinoma. J Pathol (2013) 231:311–22. doi: 10.1002/path.4239 23868181

[142] Wang LW Jiang S Gewurz BE. Epstein-Barr virus LMP1-mediated oncogenicity. J Virol (2017) 91(21):10–1128. doi: 10.1128/JVI.01718-16 PMC5640852 28835489

[143] Soni V Cahir-McFarland E Kieff E. LMP1 TRAFficking activates growth and survival pathways. Adv Exp Med Biol (2007) 597:173–87. doi: 10.1007/978-0-387-70630-6_14 17633026

[144] Wu L Nakano H Wu Z. The C-terminal activating region 2 of the Epstein-Barr virus-encoded latent membrane protein 1 activates NF-kappaB through TRAF6 and TAK1. J Biol Chem (2006) 281:2162–9. doi: 10.1074/jbc.M505903200 16280329

[145] Luftig M Yasui T Soni V Kang M-S Jacobson N Cahir-McFarland E. Epstein-Barr virus latent infection membrane protein 1 TRAF-binding site induces NIK/IKK alpha-dependent noncanonical NF-kappaB activation. Proc Natl Acad Sci USA (2004) 101:141–6. doi: 10.1073/pnas.223718310 PMC314152 14691250

[146] Sun S-C. Non-canonical NF-κB signaling pathway. Cell Res (2011) 21:71–85. doi: 10.1038/cr.2010.177 21173796 PMC3193406

[147] Kung C-P Raab-Traub N. Epstein-Barr virus latent membrane protein 1 modulates distinctive NF- kappaB pathways through C-terminus-activating region 1 to regulate epidermal growth factor receptor expression. J Virol (2010) 84:6605–14. doi: 10.1128/JVI.00344-10 PMC2903255 20410275

[148] Verhoeven RJA Tong S Zhang G Zong J Chen Y Jin D-Y. NF-κB signaling regulates expression of Epstein-Barr virus BART microRNAs and long noncoding RNAs in nasopharyngeal carcinoma. J Virol (2016) 90:6475–88. doi: 10.1128/JVI.00613-16 PMC4936125 27147748

[149] Lechner M Schartinger VH Steele CD Nei WL Ooft ML Schreiber L-M. Somatostatin receptor 2 expression in nasopharyngeal cancer is induced by Epstein Barr virus infection: impact on prognosis, imaging and therapy. Nat Commun (2021) 12:117. doi: 10.1038/s41467-020-20308-8 33402692 PMC7785735

[150] Burgos JS. Involvement of the Epstein-Barr virus in the nasopharyngeal carcinoma pathogenesis. Med Oncol (2005) 22:113–21. doi: 10.1385/MO:22:2:113 15965273

[151] Incrocci R Hussain S Stone A Bieging K Alt LAC Fay MJ. Epstein–Barr virus Latent Membrane Protein 2A (LMP2A)-mediated changes in Fas expression and Fas-dependent apoptosis: Role of Lyn/Syk activation. Cell Immunol (2015) 297:108–19. doi: 10.1016/j.cellimm.2015.08.001 PMC4618070 26255694

[152] Cen O Longnecker R. Latent membrane protein 2 (LMP2). Curr Top Microbiol Immunol (2015) 391:151–80. doi: 10.1007/978-3-319-22834-1_5 26428374

[153] Altmann M Pich D Ruiss R Wang J Sugden B Hammerschmidt W. Transcriptional activation by EBV nuclear antigen 1 is essential for the expression of EBV’s transforming genes. Proc Natl Acad Sci USA (2006) 103:14188–93. doi: 10.1073/pnas.0605985103 PMC1599932 16966603

[154] Frappier L. Role of EBNA1 in NPC tumourigenesis. Semin Cancer Biol (2012) 22:154–61. doi: 10.1016/j.semcancer.2011.12.002 22206863

[155] Lu J Murakami M Verma SC Cai Q Haldar S Kaul R. Epstein-Barr Virus nuclear antigen 1 (EBNA1) confers resistance to apoptosis in EBV-positive B-lymphoma cells through up-regulation of survivin. Virology (2011) 410:64–75. doi: 10.1016/j.virol.2010.10.029 21093004 PMC4287362

[156] Li JSZ Abbasi A Kim DH Lippman SM Alexandrov LB Cleveland DW. Chromosomal fragile site breakage by EBV-encoded EBNA1 at clustered repeats. Nature (2023) 616:504–9. doi: 10.1038/s41586-023-05923-x PMC10328181 37046091

[157] Chien YC Chen JY Liu MY Yang HI Hsu MM Chen CJ. Serologic markers of Epstein-Barr virus infection and nasopharyngeal carcinoma in Taiwanese men. N Engl J Med (2001) 345:1877–82. doi: 10.1056/NEJMoa011610 11756578

[158] Soldan SS Anderson EM Frase DM Zhang Y Caruso LB Wang Y. EBNA1 inhibitors have potent and selective antitumor activity in xenograft models of Epstein-Barr virus-associated gastric cancer. Gastric Cancer (2021) 24:1076–88. doi: 10.1007/s10120-021-01193-6 PMC8338878 33929613

[159] Zhang J Jia L Tsang CM Tsao SW. EBV infection and glucose metabolism in nasopharyngeal carcinoma. Adv Exp Med Biol (2017) 1018:75–90. doi: 10.1007/978-981-10-5765-6_6 29052133

[160] Kim H Choi H Lee SK. Epstein-Barr virus miR-BART20-5p regulates cell proliferation and apoptosis by targeting BAD. Cancer Lett (2015) 356:733–42. doi: 10.1016/j.canlet.2014.10.023 25449437

[161] Cai L-M Lyu X-M Luo W-R Cui X-F Ye Y-F Yuan C-C. EBV-miR-BART7-3p promotes the EMT and metastasis of nasopharyngeal carcinoma cells by suppressing the tumor suppressor PTEN. Oncogene (2015) 34:2156–66. doi: 10.1038/onc.2014.341 25347742

[162] Zeng Z-Y Zhou Y-H Zhang W-L Xiong W Fan S-Q Li X-L. Gene expression profiling of nasopharyngeal carcinoma reveals the abnormally regulated Wnt signaling pathway. Hum Pathol (2007) 38:120–33. doi: 10.1016/j.humpath.2006.06.023 16996564

[163] Webb N Connolly G Tellam J Yap AS Khanna R. Epstein-Barr virus associated modulation of Wnt pathway is not dependent on latent membrane protein-1. PloS One (2008) 3:e3254. doi: 10.1371/journal.pone.0003254 18806872 PMC2532746

[164] Morrison JA Raab-Traub N. Roles of the ITAM and PY motifs of Epstein-Barr virus latent membrane protein 2A in the inhibition of epithelial cell differentiation and activation of {beta}-catenin signaling. J Virol (2005) 79:2375–82. doi: 10.1128/JVI.79.4.2375-2382.2005 PMC546559 15681438

[165] Horikawa T Yoshizaki T Kondo S Furukawa M Kaizaki Y Pagano JS. Epstein-Barr Virus latent membrane protein 1 induces Snail and epithelial-mesenchymal transition in metastatic nasopharyngeal carcinoma. Br J Cancer (2011) 104:1160–7. doi: 10.1038/bjc.2011.38 PMC3068490 21386845

[166] Port RJ Pinheiro-Maia S Hu C Arrand JR Wei W Young LS. Epstein-Barr virus induction of the Hedgehog signalling pathway imposes a stem cell phenotype on human epithelial cells. J Pathol (2013) 231:367–77. doi: 10.1002/path.4245 23934731

[167] Lo AKF Dawson CW Lo KW Yu Y Young LS. Upregulation of Id1 by Epstein-Barr virus-encoded LMP1 confers resistance to TGFbeta-mediated growth inhibition. Mol Cancer (2010) 9:155. doi: 10.1186/1476-4598-9-155 20565867 PMC2908095

[168] Yao M Ohshima K Suzumiya J Kume T Shiroshita T Kikuchi M. Interleukin-10 expression and cytotoxic-T-cell response in Epstein-Barr-virus-associated nasopharyngeal carcinoma. Int J Cancer (1997) 72:398–402. doi: 10.1002/(SICI)1097-0215(19970729)72:3<398::AID-IJC4>3.0.CO;2-K 9247280

[169] Gullo C Low WK Teoh G. Association of Epstein-Barr virus with nasopharyngeal carcinoma and current status of development of cancer-derived cell lines. Ann Acad Med Singapore (2008) 37:769–77. doi: 10.47102/annals-acadmedsg.V37N9p769 18989494

[170] Johannsen E Koh E Mosialos G Tong X Kieff E Grossman SR. Epstein-Barr virus nuclear protein 2 transactivation of the latent membrane protein 1 promoter is mediated by J kappa and PU.1. J Virol (1995) 69:253–62. doi: 10.1128/jvi.69.1.253-262.1995 PMC188571 7983717

[171] O’Neil JD Owen TJ Wood VHJ Date KL Valentine R Chukwuma MB. Epstein-Barr virus-encoded EBNA1 modulates the AP-1 transcription factor pathway in nasopharyngeal carcinoma cells and enhances angiogenesis in *vitro* . J Gen Virol (2008) 89:2833–42. doi: 10.1099/vir.0.2008/003392-0 18931081

[172] Yoshizaki T Horikawa T Qing-Chun R Wakisaka N Takeshita H Sheen TS. Induction of interleukin-8 by Epstein-Barr virus latent membrane protein-1 and its correlation to angiogenesis in nasopharyngeal carcinoma. Clin Cancer Res (2001) 7:1946–51. 11448908

[173] Lyu X Wang J Guo X Wu G Jiao Y Faleti OD. EBV-miR-BART1-5P activates AMPK/mTOR/HIF1 pathway via a PTEN independent manner to promote glycolysis and angiogenesis in nasopharyngeal carcinoma. PloS Pathog (2018) 14:e1007484. doi: 10.1371/journal.ppat.1007484 30557400 PMC6312352

[174] Yuan J Zhang F Niu R. Multiple regulation pathways and pivotal biological functions of STAT3 in cancer. Sci Rep (2015) 5:17663. doi: 10.1038/srep17663 26631279 PMC4668392

[175] El-Sharkawy A Al Zaidan L Malki A. Epstein–Barr virus-associated Malignancies: roles of viral oncoproteins in carcinogenesis. Front Oncol (2018) 8:265. doi: 10.3389/fonc.2018.00265 30116721 PMC6082928

[176] Ren Q Sato H Murono S Furukawa M Yoshizaki T. Epstein-Barr virus (EBV) latent membrane protein 1 induces interleukin-8 through the nuclear factor-kappa B signaling pathway in EBV-infected nasopharyngeal carcinoma cell line. Laryngoscope (2004) 114:855–9. doi: 10.1097/00005537-200405000-00012 15126743

[177] Sheu LF Chen A Meng CL Ho KC Lee WH Leu FJ. Enhanced Malignant progression of nasopharyngeal carcinoma cells mediated by the expression of Epstein-Barr nuclear antigen 1 in *vivo* . J Pathol (1996) 180:243–8. doi: 10.1002/(SICI)1096-9896(199611)180:3<243::AID-PATH655>3.0.CO;2-7 8958799

[178] Cheng S Li Z He J Fu S Duan Y Zhou Q. Epstein–Barr virus noncoding RNAs from the extracellular vesicles of nasopharyngeal carcinoma (NPC) cells promote angiogenesis via TLR3/RIG-I-mediated VCAM-1 expression. Biochim Biophys Acta (BBA) - Mol Basis Dis (2019) 1865:1201–13. doi: 10.1016/j.bbadis.2019.01.015 30659926

[179] Du Y Zhang J-Y Gong L-P Feng Z-Y Wang D Pan Y-H. Hypoxia-induced ebv-circLMP2A promotes angiogenesis in EBV-associated gastric carcinoma through the KHSRP/VHL/HIF1α/VEGFA pathway. Cancer Lett (2022) 526:259–72. doi: 10.1016/j.canlet.2021.11.031 34863886

[180] Sunakawa Y Lenz H-J. Molecular classification of gastric adenocarcinoma: translating new insights from the cancer genome atlas research network. Curr Treat Options Oncol (2015) 16:17. doi: 10.1007/s11864-015-0331-y 25813036

[181] Liu X Wang Y Wang X Sun Z Li L Tao Q. Epigenetic silencing of WNT5A in Epstein-Barr virus-associated gastric carcinoma. Arch Virol (2013) 158:123–32. doi: 10.1007/s00705-012-1481-x 23001722

[182] Xiang T Lin Y-X Ma W Zhang H-J Chen K-M He G-P. Vasculogenic mimicry formation in EBV-associated epithelial Malignancies. Nat Commun (2018) 9:5009. doi: 10.1038/s41467-018-07308-5 30479336 PMC6258759

[183] Yoshizaki T Sato H Furukawa M Pagano JS. The expression of matrix metalloproteinase 9 is enhanced by Epstein–Barr virus latent membrane protein 1. Proc Natl Acad Sci (1998) 95:3621–6. doi: 10.1073/pnas.95.7.3621 PMC19885 9520415

[184] Horikawa T Yoshizaki T Sheen TS Lee SY Furukawa M. Association of latent membrane protein 1 and matrix metalloproteinase 9 with metastasis in nasopharyngeal carcinoma. Cancer (2000) 89:715–23. doi: 10.1002/1097-0142(20000815)89:4<715::AID-CNCR1>3.0.CO;2-9 10951332

[185] Jabłońska-Trypuć A Matejczyk M Rosochacki S. Matrix metalloproteinases (MMPs), the main extracellular matrix (ECM) enzymes in collagen degradation, as a target for anticancer drugs. J Enzyme Inhib Med Chem (2016) 31:177–83. doi: 10.3109/14756366.2016.1161620 27028474

[186] Ishiguro H Kimura M Takeyama H. Role of microRNAs in gastric cancer. World J Gastroenterol (2014) 20:5694–9. doi: 10.3748/wjg.v20.i19.5694 PMC4024779 24914330

[187] Nkosi D Sun L Duke LC Meckes DG Jr. Epstein-Barr virus LMP1 manipulates the content and functions of extracellular vesicles to enhance metastatic potential of recipient cells. PloS Pathog (2020) 16:e1009023. doi: 10.1371/journal.ppat.1009023 33382850 PMC7774862

[188] Meng D-F Xie P Peng L-X Sun R Luo D-H Chen Q-Y. CDC42-interacting protein 4 promotes metastasis of nasopharyngeal carcinoma by mediating invadopodia formation and activating EGFR signaling. J Exp Clin Cancer Res (2017) 36:21. doi: 10.1186/s13046-016-0483-z 28129778 PMC5273811

[189] Ma L Deng X Wu M Zhang G Huang J. Down-regulation of miRNA-204 by LMP-1 enhances CDC42 activity and facilitates invasion of EBV-associated nasopharyngeal carcinoma cells. FEBS Lett (2014) 588:1562–70. doi: 10.1016/j.febslet.2014.02.039 24613926

[190] Liu H-P Chen C-C Wu C-C Huang Y-C Liu S-C Liang Y. Epstein-Barr virus-encoded LMP1 interacts with FGD4 to activate Cdc42 and thereby promote migration of nasopharyngeal carcinoma cells. PloS Pathog (2012) 8:e1002690. doi: 10.1371/journal.ppat.1002690 22589722 PMC3349753

[191] Huang SCM Tsao SW Tsang CM. Interplay of viral infection, host cell factors and tumor microenvironment in the pathogenesis of nasopharyngeal carcinoma. Cancers (2018) 10(4):106. doi: 10.3390/cancers10040106 29617291 PMC5923361

[192] Kong Q-L Hu L-J Cao J-Y Huang Y-J Xu L-H Liang Y. Epstein-Barr virus-encoded LMP2A induces an epithelial-mesenchymal transition and increases the number of side population stem-like cancer cells in nasopharyngeal carcinoma. PloS Pathog (2010) 6:e1000940. doi: 10.1371/journal.ppat.1000940 20532215 PMC2880580

[193] Shinozaki A Sakatani T Ushiku T Hino R Isogai M Ishikawa S. Downregulation of microRNA-200 in EBV-associated gastric carcinoma. Cancer Res (2010) 70:4719–27. doi: 10.1158/0008-5472.CAN-09-4620 20484038

[194] Tsai C-Y Liu YY Liu K-H Hsu J-T Chen T-C Chiu C-T. Comprehensive profiling of virus microRNAs of Epstein-Barr virus-associated gastric carcinoma: highlighting the interactions of ebv-Bart9 and host tumor cells. J Gastroenterol Hepatol (2017) 32:82–91. doi: 10.1111/jgh.13432 27144885

[195] Hanahan D Weinberg RA. Hallmarks of cancer: the next generation. Cell (2011) 144:646–74. doi: 10.1016/j.cell.2011.02.013 21376230

[196] Lo AK-F Dawson CW Young LS Ko C-W Hau P-M Lo K-W. Activation of the FGFR1 signalling pathway by the Epstein-Barr virus-encoded LMP1 promotes aerobic glycolysis and transformation of human nasopharyngeal epithelial cells. J Pathol (2015) 237:238–48. doi: 10.1002/path.4575 26096068

[197] Zhang J Jia L Liu T Yip YL Tang WC Lin W. mTORC2-mediated PDHE1α nuclear translocation links EBV-LMP1 reprogrammed glucose metabolism to cancer metastasis in nasopharyngeal carcinoma. Oncogene (2019) 38:4669–84. doi: 10.1038/s41388-019-0749-y PMC6756087 30745576

[198] Cai T-T Ye S-B Liu Y-N He J Chen Q-Y Mai H-Q. LMP1-mediated glycolysis induces myeloid-derived suppressor cell expansion in nasopharyngeal carcinoma. PloS Pathog (2017) 13:e1006503. doi: 10.1371/journal.ppat.1006503 28732079 PMC5540616

[199] Lo AK-F Dawson CW Lung HL Wong K-L Young LS. The role of EBV-encoded LMP1 in the NPC tumor microenvironment: from function to therapy. Front Oncol (2021) 11:640207. doi: 10.3389/fonc.2021.640207 33718235 PMC7947715

[200] Mainou BA Everly DN Jr Raab-Traub N. Unique signaling properties of CTAR1 in LMP1-mediated transformation. J Virol (2007) 81:9680–92. doi: 10.1128/JVI.01001-07 PMC2045399 17626074

[201] Yin H Qu J Peng Q Gan R. Molecular mechanisms of EBV-driven cell cycle progression and oncogenesis. Med Microbiol Immunol (2019) 208:573–83. doi: 10.1007/s00430-018-0570-1 PMC6746687 30386928

[202] Ding L Li L Yang J Zhou S Li W Tang M. Latent membrane protein 1 encoded by Epstein-Barr virus induces telomerase activity via p16INK4A/Rb/E2F1 and JNK signaling pathways. J Med Virol (2007) 79:1153–63. doi: 10.1002/jmv.20896 17597480

[203] Yang X He Z Xin B Cao L. LMP1 of Epstein–Barr virus suppresses cellular senescence associated with the inhibition of p16INK4a expression. Oncogene (2000) 19:2002–13. doi: 10.1038/sj.onc.1203515 10803461

[204] Li S Du H Wang Z Zhou L Zhao X Zeng Y. Meta-analysis of the relationship between Epstein-Barr virus infection and clinicopathological features of patients with gastric carcinoma. Sci China Life Sci (2010) 53:524–30. doi: 10.1007/s11427-010-0082-8 20596921

[205] Liu X Gao Y Luo B Zhao Y. Construction and antiapoptosis activities of recombinant adenoviral expression vector carrying EBV latent membrane protein 2A. Gastroenterol Res Pract (2011) 2011:182832. doi: 10.1155/2011/182832 21860618 PMC3157153

[206] Fukuda M Longnecker R. Latent membrane protein 2A inhibits transforming growth factor-beta 1-induced apoptosis through the phosphatidylinositol 3-kinase/Akt pathway. J Virol (2004) 78:1697–705. doi: 10.1128/JVI.78.4.1697-1705.2004 PMC369507 14747535

[207] Fukuda M Longnecker R. Epstein-Barr virus latent membrane protein 2A mediates transformation through constitutive activation of the Ras/PI3-K/Akt Pathway. J Virol (2007) 81:9299–306. doi: 10.1128/JVI.00537-07 PMC1951437 17582000

[208] Marquitz AR Mathur A Nam CS Raab-Traub N. The Epstein–Barr Virus BART microRNAs target the pro-apoptotic protein Bim. Virology (2011) 412:392–400. doi: 10.1016/j.virol.2011.01.028 21333317 PMC3340891

[209] Tsang CM Yip YL Lo KW Deng W To KF Hau PM. Cyclin D1 overexpression supports stable EBV infection in nasopharyngeal epithelial cells. Proc Natl Acad Sci U.S.A. (2012) 109:E3473–82. doi: 10.1073/pnas.1202637109 PMC3528537 23161911

[210] Ohtani N Brennan P Gaubatz S Sanij E Hertzog P Wolvetang E. Epstein-Barr virus LMP1 blocks p16INK4a-RB pathway by promoting nuclear export of E2F4/5. J Cell Biol (2003) 162:173–83. doi: 10.1083/jcb.200302085 PMC2172795 12860972

[211] Chen C Lu Z Yang J Hao W Qin Y Wang H. MiR-17-5p promotes cancer cell proliferation and tumorigenesis in nasopharyngeal carcinoma by targeting p21. Cancer Med (2016) 5:3489–99. doi: 10.1002/cam4.863 PMC5224848 27774777

[212] Yuan L Li S Chen Q Xia T Luo D Li L. EBV infection-induced GPX4 promotes chemoresistance and tumor progression in nasopharyngeal carcinoma. Cell Death Differ (2022) 29:1513–27. doi: 10.1038/s41418-022-00939-8 PMC9346003 35105963

[213] Shi F Zhou M Shang L Du Q Li Y Xie L. EBV(LMP1)-induced metabolic reprogramming inhibits necroptosis through the hypermethylation of the RIP3 promoter. Theranostics (2019) 9:2424–38. doi: 10.7150/thno.30941 PMC6525991 31131045

[214] Effert P McCoy R Abdel-Hamid M Flynn K Zhang Q Busson P. Alterations of the p53 gene in nasopharyngeal carcinoma. J Virol (1992) 66:3768–75. doi: 10.1128/jvi.66.6.3768-3775.1992 PMC241162 1349927

[215] Spruck CH 3rd Tsai YC Huang DP Yang AS Rideout WM 3rd Gonzalez-Zulueta M. Absence of p53 gene mutations in primary nasopharyngeal carcinomas. Cancer Res (1992) 52:4787–90. 1511442

[216] Lo KW Mok CH Huang DP Liu YX Choi PH Lee JC. p53 mutation in human nasopharyngeal carcinomas. Anticancer Res (1992) 12:1957–63. 1295443

[217] Wu H-C Lu T-Y Lee J-J Hwang J-K Lin Y-J Wang C-K. MDM2 expression in EBV-infected nasopharyngeal carcinoma cells. Lab Invest (2004) 84:1547–56. doi: 10.1038/labinvest.3700183 15448710

[218] Li J Zhang Y Sun L Liu S Zhao M Luo B. LMP1 Induces p53 Protein Expression via the H19/miR-675-5p Axis. Microbiol Spectr (2022) 10:e0000622. doi: 10.1128/spectrum.00006-22 35674441 PMC9241841

[219] Zheng X Wang J Wei L Peng Q Gao Y Fu Y. Epstein-Barr Virus MicroRNA miR-BART5-3p Inhibits p53 Expression. J Virol (2018) 92(23):10–1128. doi: 10.1128/JVI.01022-18 PMC6232473 30209170

[220] Crook T Nicholls JM Brooks L O’Nions J Allday MJ. High level expression of deltaN-p63: a mechanism for the inactivation of p53 in undifferentiated nasopharyngeal carcinoma (NPC)? Oncogene (2000) 19:3439–44. doi: 10.1038/sj.onc.1203656 10918601

[221] Zhang Y Xiong Y Yarbrough WG. ARF promotes MDM2 degradation and stabilizes p53: ARF-INK4a locus deletion impairs both the Rb and p53 tumor suppression pathways. Cell (1998) 92:725–34. doi: 10.1016/S0092-8674(00)81401-4 9529249

[222] Ribeiro J Malta M Galaghar A Silva F Afonso LP Medeiros R. P53 deregulation in Epstein-Barr virus-associated gastric cancer. Cancer Lett (2017) 404:37–43. doi: 10.1016/j.canlet.2017.07.010 28729047

[223] Wang J Zheng X Qin Z Wei L Lu Y Peng Q. Epstein-Barr virus miR-BART3-3p promotes tumorigenesis by regulating the senescence pathway in gastric cancer. J Biol Chem (2019) 294:4854–66. doi: 10.1074/jbc.RA118.006853 PMC6442059 30674552

[224] Huang S Shu L Dilling MB Easton J Harwood FC Ichijo H. Sustained activation of the JNK cascade and rapamycin-induced apoptosis are suppressed by p53/p21Cip1. Mol Cell (2003) 11:1491–501. doi: 10.1016/S1097-2765(03)00180-1 12820963

[225] Potapova O Gorospe M Dougherty RH Dean NM Gaarde WA Holbrook NJ. Inhibition of c-Jun N-terminal kinase 2 expression suppresses growth and induces apoptosis of human tumor cells in a p53-dependent manner. Mol Cell Biol (2000) 20:1713–22. doi: 10.1128/MCB.20.5.1713-1722.2000 PMC85354 10669748

[226] Yoshizaki T Ito M Murono S Wakisaka N Kondo S Endo K. Current understanding and management of nasopharyngeal carcinoma. Auris Nasus Larynx (2012) 39:137–44. doi: 10.1016/j.anl.2011.02.012 21592702

[227] Binnewies M Roberts EW Kersten K Chan V Fearon DF Merad M. Understanding the tumor immune microenvironment (TIME) for effective therapy. Nat Med (2018) 24:541–50. doi: 10.1038/s41591-018-0014-x PMC5998822 29686425

[228] Pai S O’Sullivan B Abdul-Jabbar I Peng J Connoly G Khanna R. Nasopharyngeal carcinoma-associated Epstein-Barr virus-encoded oncogene latent membrane protein 1 potentiates regulatory T-cell function. Immunol Cell Biol (2007) 85:370–7. doi: 10.1038/sj.icb.7100046 17372611

[229] Huo S Luo Y Deng R Liu X Wang J Wang L. EBV-EBNA1 constructs an immunosuppressive microenvironment for nasopharyngeal carcinoma by promoting the chemoattraction of Treg cells. J Immunother Cancer (2020) 8(2). doi: 10.1136/jitc-2020-001588 PMC7597532 33122398

[230] Wang J Luo Y Bi P Lu J Wang F Liu X. Mechanisms of Epstein-Barr virus nuclear antigen 1 favor Tregs accumulation in nasopharyngeal carcinoma. Cancer Med (2020) 9:5598–608. doi: 10.1002/cam4.3213 PMC7402843 32573058

[231] Yip WK Abdullah MA Yusoff SM Seow HF. Increase in tumour-infiltrating lymphocytes with regulatory T cell immunophenotypes and reduced zeta-chain expression in nasopharyngeal carcinoma patients. Clin Exp Immunol (2009) 155:412–22. doi: 10.1111/j.1365-2249.2008.03793.x PMC2669517 19220831

[232] Ma J Li J Hao Y Nie Y Li Z Qian M. Differentiated tumor immune microenvironment of Epstein-Barr virus-associated and negative gastric cancer: implication in prognosis and immunotherapy. Oncotarget (2017) 8:67094–103. doi: 10.18632/oncotarget.17945 PMC5620158 28978018

[233] Gong L-P Chen J-N Xiao L He Q Feng Z-Y Zhang Z-G. The implication of tumor-infiltrating lymphocytes in Epstein-Barr virus–associated gastric carcinoma. Hum Pathol (2019) 85:82–91. doi: 10.1016/j.humpath.2018.11.002 30448221

[234] Strong MJ Xu G Coco J Baribault C Vinay DS Lacey MR. Differences in gastric carcinoma microenvironment stratify according to EBV infection intensity: implications for possible immune adjuvant therapy. PloS Pathog (2013) 9:e1003341. doi: 10.1371/journal.ppat.1003341 23671415 PMC3649992

[235] Wang J Ge J Wang Y Xiong F Guo J Jiang X. EBV miRNAs BART11 and BART17-3p promote immune escape through the enhancer-mediated transcription of PD-L1. Nat Commun (2022) 13:866. doi: 10.1038/s41467-022-28479-2 35165282 PMC8844414

[236] Ge J Wang J Xiong F Jiang X Zhu K Wang Y. Epstein-Barr virus-encoded circular RNA circBART2.2 promotes immune escape of nasopharyngeal carcinoma by regulating PD-L1. Cancer Res (2021) 81:5074–88. doi: 10.1158/0008-5472.CAN-20-4321 PMC8974435 34321242

[237] Derks S Liao X Chiaravalli AM Xu X Camargo MC Solcia E. Abundant PD-L1 expression in Epstein-Barr Virus-infected gastric cancers. Oncotarget (2016) 7:32925–32. doi: 10.18632/oncotarget.9076 PMC5078063 27147580

[238] Cho J Kang M-S Kim K-M. Epstein-Barr virus-associated gastric carcinoma and specific features of the accompanying immune response. J Gastric Cancer (2016) 16:1–7. doi: 10.5230/jgc.2016.16.1.1 27104020 PMC4834615

[239] Lu S Wang LJ Lombardo K Kwak Y Kim WH Resnick MB. Expression of indoleamine 2, 3-dioxygenase 1 (IDO1) and tryptophanyl-tRNA synthetase (WARS) in gastric cancer molecular subtypes. Appl Immunohistochem Mol Morphol (2020) 28:360–8. doi: 10.1097/PAI.0000000000000761 PMC6813876 31033497

[240] Zhang NN Chen JN Xiao L Tang F Zhang ZG Zhang YW. Accumulation mechanisms of CD4 (+) CD25 (+) FOXP3 (+) regulatory T cells in EBV-associated gastric carcinoma. Sci Rep (2015) 5:18057. doi: 10.1038/srep18057 26673957 PMC4682180

[241] Chen J-N Jiang Y Li H-G Ding Y-G Fan X-J Xiao L. Epstein-Barr virus genome polymorphisms of Epstein-Barr virus-associated gastric carcinoma in gastric remnant carcinoma in Guangzhou, southern China, an endemic area of nasopharyngeal carcinoma. Virus Res (2011) 160:191–9. doi: 10.1016/j.virusres.2011.06.011 21723347

[242] Nasopharyngeal cancer treatment options. Available at: https://www.cancer.org/cancer/nasopharyngeal-cancer/treating/by-stage.html (Accessed March 23, 2023).

[243] Chen Y-P Liu X Zhou Q Yang K-Y Jin F Zhu X-D. Metronomic capecitabine as adjuvant therapy in locoregionally advanced nasopharyngeal carcinoma: a multicentre, open-label, parallel-group, randomised, controlled, phase 3 trial. Lancet (2021) 398:303–13. doi: 10.1016/S0140-6736(21)01123-5 34111416

[244] Lin Z Fu S Zhou Y Zhang X Chen C He L-N. First-line platinum-based chemotherapy and survival outcomes in locally advanced or metastatic pulmonary lymphoepithelioma-like carcinoma. Lung Cancer (2019) 137:100–7. doi: 10.1016/j.lungcan.2019.09.007 31568886

[245] Bao H Ma LZ Zhao C Yu M Zhang B Zhang J. Anti-angiogenic therapy for advanced primary pulmonary lymphoepithelioma-like carcinoma: a retrospective multicenter study. J Cancer Res Clin Oncol (2023) 149:1185–93. doi: 10.1007/s00432-022-03935-0 PMC9984323 35377040

[246] Narayanan A Knollmann FD Walby JAS Lim S Gandara DR Riess JW. EBV-positive primary pulmonary lymphoepithelioma-like carcinoma response to PD-L1 blockade. Clin Lung Cancer (2019) 20:e238–41. doi: 10.1016/j.cllc.2018.12.015 30679078

[247] Nishikawa J Iizasa H Yoshiyama H Shimokuri K Kobayashi Y Sasaki S. Clinical importance of Epstein^–^Barr virus-associated gastric cancer. Cancers (2018) 10(6):167. doi: 10.3390/cancers10060167 29843478 PMC6024931

[248] Jain A Chia WK Toh HC. Immunotherapy for nasopharyngeal cancer-a review. Chin Clin Oncol (2016) 5:22. doi: 10.21037/cco.2016.03.08 27121882

[249] Ma BBY Lim W-T Goh B-C Hui EP Lo K-W Pettinger A. Antitumor activity of nivolumab in recurrent and metastatic nasopharyngeal carcinoma: an international, multicenter study of the mayo clinic phase 2 consortium (NCI-9742). J Clin Oncol (2018) 36:1412–8. doi: 10.1200/JCO.2017.77.0388 PMC5941615 29584545

[250] Hsu C Lee S-H Ejadi S Even C Cohen RB Le Tourneau C. Safety and antitumor activity of pembrolizumab in patients with programmed death-ligand 1-positive nasopharyngeal carcinoma: results of the KEYNOTE-028 study. J Clin Oncol (2017) 35:4050–6. doi: 10.1200/JCO.2017.73.3675 28837405

[251] Xu R-H Mai H-Q Chen Q-Y Chen D Hu C Yang K. JUPITER-02: Randomized, double-blind, phase III study of toripalimab or placebo plus gemcitabine and cisplatin as first-line treatment for recurrent or metastatic nasopharyngeal carcinoma (NPC). J Clin Orthod (2021) 39:LBA2–2. doi: 10.1200/JCO.2021.39.15_suppl.LBA2

[252] Yang Y Qu S Li J Hu C Xu M Li W. Camrelizumab versus placebo in combination with gemcitabine and cisplatin as first-line treatment for recurrent or metastatic nasopharyngeal carcinoma (CAPTAIN-1st): a multicentre, randomised, double-blind, phase 3 trial. Lancet Oncol (2021) 22:1162–74. doi: 10.1016/S1470-2045(21)00302-8 34174189

[253] Zhang L Yang Y Pan J-J Chen X Sun Y Wang H. RATIONALE-309: Updated progression-free survival (PFS), PFS after next line of treatment, and overall survival from a phase 3 double-blind trial of tislelizumab versus placebo, plus chemotherapy, as first-line treatment for recurrent/metastatic nasopharyngeal cancer. J Clin Orthod (2022) 40:384950–0. doi: 10.1200/JCO.2022.40.36_suppl.384950

[254] Chan ATC Lee VHF Hong R-L Ahn M-J Chong WQ Kim S-B. Pembrolizumab monotherapy versus chemotherapy in platinum-pretreated, recurrent or metastatic nasopharyngeal cancer (KEYNOTE-122): an open-label, randomized, phase III trial. Ann Oncol (2023) 34:251–61. doi: 10.1016/j.annonc.2022.12.007 36535566

[255] Wu Z Xian X Wang K Cheng D Li W Chen B. Immune checkpoint blockade therapy may be a feasible option for primary pulmonary lymphoepithelioma-like carcinoma. Front Oncol (2021) 11:626566. doi: 10.3389/fonc.2021.626566 33981599 PMC8110193

[256] Tang L Chen N He W Zhou J Zhang J Lin Z. The clinicopathological features and prognosis of primary pulmonary lymphoepithelioma-like carcinoma: A systematic review and meta-analysis. PloS One (2020) 15:e0240729. doi: 10.1371/journal.pone.0240729 33064745 PMC7567369

[257] Zhong Y-M Yin K Chen Y Xie Z Lv Z-Y Yang J-J. PD-1/PD-L1 combined with LAG3 is associated with clinical activity of immune checkpoint inhibitors in metastatic primary pulmonary lymphoepithelioma-like carcinoma. Front Immunol (2022) 13:951817. doi: 10.3389/fimmu.2022.951817 36263036 PMC9574915

[258] Kim ST Cristescu R Bass AJ Kim K-M Odegaard JI Kim K. Comprehensive molecular characterization of clinical responses to PD-1 inhibition in metastatic gastric cancer. Nat Med (2018) 24:1449–58. doi: 10.1038/s41591-018-0101-z 30013197

[259] Janjigian YY Shitara K Moehler M Garrido M Salman P Shen L. First-line nivolumab plus chemotherapy versus chemotherapy alone for advanced gastric, gastro-oesophageal junction, and oesophageal adenocarcinoma (CheckMate 649): a randomised, open-label, phase 3 trial. Lancet (2021) 398:27–40. doi: 10.1016/S0140-6736(21)00797-2 34102137 PMC8436782

[260] Chia W-K Teo M Wang W-W Lee B Ang S-F Tai W-M. Adoptive T-cell transfer and chemotherapy in the first-line treatment of metastatic and/or locally recurrent nasopharyngeal carcinoma. Mol Ther (2014) 22:132–9. doi: 10.1038/mt.2013.242 PMC3978790 24297049

[261] Toh HC Yang M-H Wang H-M Hsieh C-Y Chitapanarux I Ho KF. 652O Randomized phase III VANCE study: Gemcitabine and carboplatin (GC) followed by Epstein Barr virus-specific autologous cytotoxic T lymphocytes (EBV-CTL) versus the same chemotherapy as first-line treatment for advanced nasopharyngeal carcinoma (NPC). Ann Oncol (2022) 33:S840. doi: 10.1016/j.annonc.2022.07.776 39241963

[262] Ye W Liu R Pan C Jiang W Zhang L Guan Z. Multicenter randomized phase 2 clinical trial of a recombinant human endostatin adenovirus in patients with advanced head and neck carcinoma. Mol Ther (2014) 22:1221–9. doi: 10.1038/mt.2014.53 PMC4048902 24662947

[263] Chen Z Xu X-H. Combining antiangiogenic therapy and radiation in nasopharyngeal carcinoma. Saudi Med J (2015) 36:659–64. doi: 10.15537/smj.2015.6.11460 PMC4454898 25987106

[264] Chia MC Shi W Li J-H Sanchez O Strathdee CA Huang D. A conditionally replicating adenovirus for nasopharyngeal carcinoma gene therapy. Mol Ther (2004) 9:804–17. doi: 10.1016/j.ymthe.2004.03.016 15194047

[265] Mocanu JD Yip KW Alajez NM Shi W Li J-H Lunt SJ. Imaging the modulation of adenoviral kinetics and biodistribution for cancer gene therapy. Mol Ther (2007) 15:921–9. doi: 10.1038/mt.sj.6300119 17356543

[266] Li N Thompson S Schultz DC Zhu W Jiang H Luo C. Discovery of selective inhibitors against EBNA1 via high throughput in silico virtual screening. PloS One (2010) 5:e10126. doi: 10.1371/journal.pone.0010126 20405039 PMC2853575

[267] Thompson S Messick T Schultz DC Reichman M Lieberman PM. Development of a high-throughput screen for inhibitors of Epstein-Barr virus EBNA1. J Biomol Screen (2010) 15:1107–15. doi: 10.1177/1087057110379154 PMC3310380 20930215

[268] Messick TE Smith GR Soldan SS McDonnell ME Deakyne JS Malecka KA. Structure-based design of small-molecule inhibitors of EBNA1 DNA binding blocks Epstein-Barr virus latent infection and tumor growth. Sci Transl Med (2019) 11(482):eaau5612. doi: 10.1126/scitranslmed.aau5612 30842315 PMC6936217

[269] Phase 1/2a Study of VK-2019 in Patients With Epstein-Barr Virus (EBV)-Positive Nasopharyngeal Carcinoma (NPC). Available at: https://clinicaltrials.gov/ct2/show/NCT03682055 (Accessed June 20, 2023).

[270] Cao Y Yang L Jiang W Wang X Liao W Tan G. Therapeutic evaluation of Epstein-Barr virus-encoded latent membrane protein-1 targeted DNAzyme for treating of nasopharyngeal carcinomas. Mol Ther (2014) 22:371–7. doi: 10.1038/mt.2013.257 PMC3916047 24322331

[271] Moore SM Cannon JS Tanhehco YC Hamzeh FM Ambinder RF. Induction of Epstein-Barr virus kinases to sensitize tumor cells to nucleoside analogues. Antimicrob Agents Chemother (2001) 45:2082–91. doi: 10.1128/AAC.45.7.2082-2091.2001 PMC90604 11408227

[272] Hislop AD Taylor GS Sauce D Rickinson AB. Cellular responses to viral infection in humans: lessons from Epstein-Barr virus. Annu Rev Immunol (2007) 25:587–617. doi: 10.1146/annurev.immunol.25.022106.141553 17378764

[273] Wang H Zhao Y Zeng L Tang M El-Deeb A Li JJ. BZLF1 controlled by family repeat domain induces lytic cytotoxicity in Epstein-Barr virus-positive tumor cells. Anticancer Res (2004) 24:67–74. 15015577

[274] Lin W Yip YL Jia L Deng W Zheng H Dai W. Establishment and characterization of new tumor xenografts and cancer cell lines from EBV-positive nasopharyngeal carcinoma. Nat Commun (2018) 9:4663. doi: 10.1038/s41467-018-06889-5 30405107 PMC6220246

[275] Hui KF Lam BHW Ho DN Tsao SW Chiang AKS. Bortezomib and SAHA synergistically induce ROS-driven caspase-dependent apoptosis of nasopharyngeal carcinoma and block replication of Epstein-Barr virus. Mol Cancer Ther (2013) 12:747–58. doi: 10.1158/1535-7163.MCT-12-0811 23475956

[276] Hui KF Ho DN Tsang CM Middeldorp JM Tsao GSW Chiang AKS. Activation of lytic cycle of Epstein-Barr virus by suberoylanilide hydroxamic acid leads to apoptosis and tumor growth suppression of nasopharyngeal carcinoma. Int J Cancer (2012) 131:1930–40. doi: 10.1002/ijc.27439 22261816

[277] Fang C-Y Lee C-H Wu C-C Chang Y-T Yu S-L Chou S-P. Recurrent chemical reactivations of EBV promotes genome instability and enhances tumor progression of nasopharyngeal carcinoma cells. Int J Cancer (2009) 124:2016–25. doi: 10.1002/ijc.24179 19132751

[278] Wildeman MA Novalic Z Verkuijlen SAWM Juwana H Huitema ADR Tan IB. Cytolytic virus activation therapy for Epstein-Barr virus-driven tumors. Clin Cancer Res (2012) 18:5061–70. doi: 10.1158/1078-0432.CCR-12-0574 22761471

[279] Li YY Chung GTY Lui VWY To K-F Ma BBY Chow C. Exome and genome sequencing of nasopharynx cancer identifies NF-κB pathway activating mutations. Nat Commun (2017) 8:14121. doi: 10.1038/ncomms14121 28098136 PMC5253631

[280] Chung A-K OuYang C-N Liu H Chao M Luo J-D Lee C-Y. Targeted sequencing of cancer-related genes in nasopharyngeal carcinoma identifies mutations in the TGF-β pathway. Cancer Med (2019) 8:5116–27. doi: 10.1002/cam4.2429 PMC6718742 31328403

[281] Chan KCA Hung ECW Woo JKS Chan PKS Leung S-F Lai FPT. Early detection of nasopharyngeal carcinoma by plasma Epstein-Barr virus DNA analysis in a surveillance program. Cancer (2013) 119:1838–44. doi: 10.1002/cncr.28001 23436393

[282] Zhang Y Tang L-L Li Y-Q Liu X Liu Q Ma J. Spontaneous remission of residual post-therapy plasma Epstein-Barr virus DNA and its prognostic implication in nasopharyngeal carcinoma: A large-scale, big-data intelligence platform-based analysis. Int J Cancer (2019) 144:2313–9. doi: 10.1002/ijc.32021 30485420

[283] Chan ATC Lo YMD Zee B Chan LYS Ma BBY Leung S-F. Plasma Epstein-Barr virus DNA and residual disease after radiotherapy for undifferentiated nasopharyngeal carcinoma. J Natl Cancer Inst (2002) 94:1614–9. doi: 10.1093/jnci/94.21.1614 12419787

[284] Li W Duan X Chen X Zhan M Peng H Meng Y. Immunotherapeutic approaches in EBV-associated nasopharyngeal carcinoma. Front Immunol (2022) 13:1079515. doi: 10.3389/fimmu.2022.1079515 36713430 PMC9875085

[285] Pender MP Csurhes PA Smith C Douglas NL Neller MA Matthews KK. Epstein-Barr virus-specific T cell therapy for progressive multiple sclerosis. JCI Insight (2018) 3(22). doi: 10.1172/jci.insight.124714 PMC6302936 30429369

[286] Li Z Zong YS. Review of the histological classification of nasopharyngeal carcinoma. J Nasopharyng Carcinoma (2014) 1:e15. doi: 10.15383/jnpc.15

[287] Morales-Sanchez A Fuentes-Panana EM. Epstein-Barr virus-associated gastric cancer and potential mechanisms of oncogenesis. Curr Cancer Drug Targets (2017) 17:534–54. doi: 10.2174/1568009616666160926124923 27677953

[288] Rowe M Rowe DT Gregory CD Young LS Farrell PJ Rupani H. Differences in B cell growth phenotype reflect novel patterns of Epstein-Barr virus latent gene expression in Burkitt’s lymphoma cells. EMBO J (1987) 6:2743–51. doi: 10.1002/j.1460-2075.1987.tb02568.x PMC553698 2824192

[289] Tsang CM Tsao SW. The role of Epstein-Barr virus infection in the pathogenesis of nasopharyngeal carcinoma. Virol Sin (2015) 30:107–21. doi: 10.1007/s12250-015-3592-5 PMC8200872 25910483

